# Design, Synthesis and Docking Studies of Flavokawain B Type Chalcones and Their Cytotoxic Effects on MCF-7 and MDA-MB-231 Cell Lines

**DOI:** 10.3390/molecules23030616

**Published:** 2018-03-08

**Authors:** Addila Abu Bakar, Muhammad Nadeem Akhtar, Norlaily Mohd Ali, Swee Keong Yeap, Ching Kheng Quah, Wan-Sin Loh, Noorjahan Banu Alitheen, Seema Zareen, Zaheer Ul-Haq, Syed Adnan Ali Shah

**Affiliations:** 1 Faculty of Industrial Sciences & Technology, University Malaysia Pahang, Lebuhraya Tun Razak, Kuantan 26300, Malaysia; addilaabubakar@gmail.com (A.A.B.); seema@ump.edu.my (S.Z.); 2 Faculty of Medicine and Health Sciences, University Tunku Abdul Rahman, Sungai Long 43400, Malaysia; norlailyma@gmail.com; 3 Chine-ASEAN College of Marine Sciences, Xiamen University Malaysia, Jalan Sunsuria, Bandar Sunsuria, Sepang 43900, Malaysia; skyeap2005@gmail.com; 4 X-ray Crystallography Unit, School of Physics, University Sains Malaysia, Penang 11800, Malaysia; ck.quah@hotmail.com (C.K.Q.); wansin_loh@live.com (W.-S.L.); 5 Department of Cell and Molecular Biology, Faculty of Biotechnology and Biomolecular Science, University Putra Malaysia, Serdang, Selangor Darul Ehsan 43400, Malaysia; 6 Dr. Panjwani Center for Molecular Medicine and Drug Research, International Center for Chemical and Biological Sciences, University of Karachi, Karachi 75270, Pakistan; zaheer.qasmi@iccs.edu; 7 Research Institute of Natural Products for Drug Discovery, Faculty of Pharmacy, University Teknologi MARA, Puncak Alam Campus, Bandar Puncak Alam 42300, Malaysia; benzene301@yahoo.com

**Keywords:** chalcone synthesis, breast cancer cell lines, SARs, anti-cancer, flavokawain B derivatives

## Abstract

Flavokawain B (**1**) is a natural chalcone extracted from the roots of *Piper methysticum,* and has been proven to be a potential cytotoxic compound. Using the partial structure of flavokawain B (FKB), about 23 analogs have been synthesized. Among them, compounds **8**, **13** and **23** were found in new FKB derivatives. All compounds were evaluated for their cytotoxic properties against two breast cancer cell lines, MCF-7 and MDA-MB-231, thus establishing the structure–activity relationship. The FKB derivatives **16** (IC_50_ = 6.50 ± 0.40 and 4.12 ± 0.20 μg/mL), **15** (IC_50_ = 5.50 ± 0.35 and 6.50 ± 1.40 μg/mL) and **13** (IC_50_ = 7.12 ± 0.80 and 4.04 ± 0.30 μg/mL) exhibited potential cytotoxic effects on the MCF-7 and MDA-MB-231 cell lines. However, the methoxy group substituted in position three and four in compound **2** (IC_50_ = 8.90 ± 0.60 and 6.80 ± 0.35 μg/mL) and **22** (IC_50_ = 8.80 ± 0.35 and 14.16 ± 1.10 μg/mL) exhibited good cytotoxicity. The lead compound FKB (**1**) showed potential cytotoxicity (IC_50_ = 7.70 ± 0.30 and 5.90 ± 0.30 μg/mL) against two proposed breast cancer cell lines. It is evident that the FKB skeleton is unique for anticancer agents, additionally, the presence of halogens (Cl and F) in position 2 and 3 also improved the cytotoxicity in FKB series. These findings could help to improve the future drug discovery process to treat breast cancer. A molecular dynamics study of active compounds revealed stable interactions within the active site of Janus kinase. The structures of all compounds were determined by ^1^H-NMR, EI-MS, IR and UV and X-ray crystallographic spectroscopy techniques.

## 1. Introduction

Breast cancer (BC) is a complex disease, composed of several subtype receptors both at the molecular and clinical level. BC is still the leading causes of death globally in women [1,2,3]. Chalcones are α,β-unsaturated carbonyl compounds with two aromatic rings (ring A and B), conjugated double bonds and a completely delocalized π-electron system [4,5]. Chalcones and their related analogues display several pharmacological properties, such as anticancer [6,7,8,9,10], anti-inflammatory [11,12,13,14], antimalarial [15,16], antileishmanial [17,18], antimicrobial [19], antifungal [20], antioxidant [21,22,23] and anticonvulsant activities [24]. Chalcone derivatives show acetylcholinesterase, tyrosinase [25,26], and monoamine oxidase (MAO) [27] enzyme inhibitory activities. Chalcones have gained the strong attention from scientists searching for new pharmacological active analogs.

Flavokawain B (FKB) (**1**), is a main bioactive chalcone isolated from the roots of *Piper methysticum* [28], which exhibited strong cytotoxicity against various cancer cell lines [29,30,31]. In this study, we synthesized **1**–**23** FKB analogs by manipulating ring B with the various substituted benzaldehydes. The main purpose when designing this proposal was to observe the effects of ring B in the breast cancer cell lines and to establish the structure–activity relationship, to eventually help future drug discovery. Previously, we investigated the antinociceptive, cytotoxic and anti-inflammatory activities of FKB (**1**) in vitro and in vivo [32,33,34,35,36].

## 2. Results and Discussion

### 2.1. Chemistry

**Figure 1:**
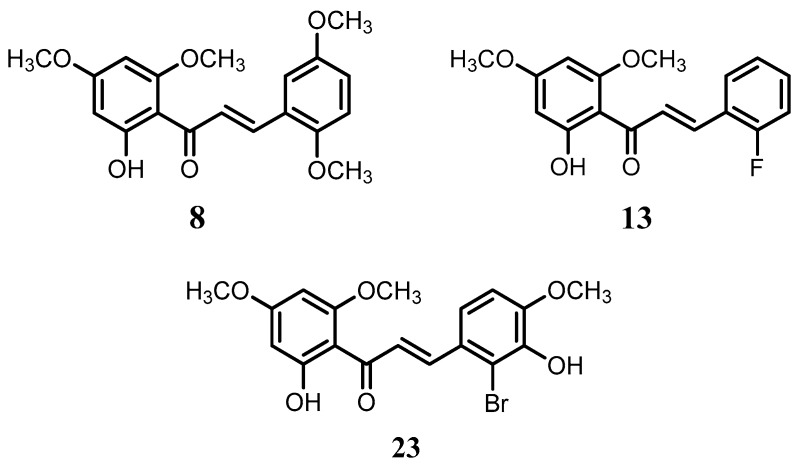
The structure of compounds **8**, **13** and **23**.

**Figure 2:**
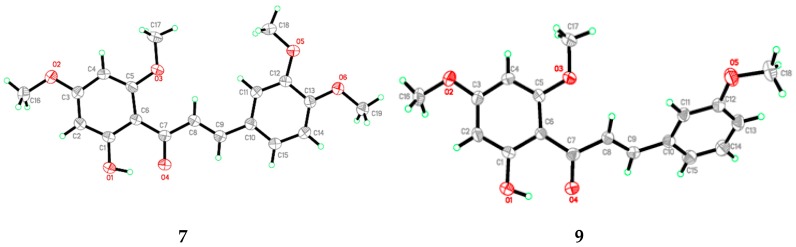
ORTEP diagram of compound **7** and **9**.

**Scheme 1:**
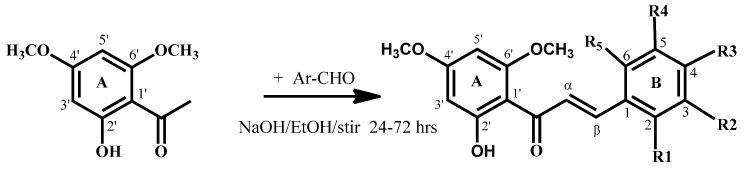
Synthesis of flavokawain B derivatives **1**–**23**.

Flavokawain B derivatives (**1**–**23**) were synthesized by Claisen–Schmidt condensation reaction using the template of FKB (**1**) (Scheme 1). FKB comprises ring A (2′-hydroxy-4′,6′-dimethoxyacetophenone) and B (benzaldehyde). Among FKB derivatives, three chalcones (*E*)-3-(2,5-dimethoxyphenyl)-1-(2′-hydroxy-4′,6′-dimethoxyphenyl)prop-2-en-1-one (**8**), *(E*)-3-(2-fluorophenyl)-1-(2′-hydroxy-4′,6′-dimethoxyphenyl)prop-2-en-1-one (**13**) and *(E*)-3-(2-bromo-3-hydroxy-4-methoxyphenyl)-1-(2′-hydroxy-4′,6′-dimethoxyphenyl)prop-2-en-1-one (**23**) were found to be new FKB analogs (Figure 1). Previously, compound **13** was synthesized by Bandgar and used as an intermediate for the preparation of nitrogen-containing chalcones, however, the data were not published [37]. The structure of FKB derivatives (**1**–**23**) were characterized by detailed spectroscopic techniques. The presence of characteristic signals appeared in ^1^H-NMR at *δ* 7.66–7.84 (*J* = 15–16 Hz) and 7.86–8.0 (*J* = 15–16 Hz) were assigned to α and β protons, respectively. The presence of carbonyl groups was supported by the signals at *δ* 191–193.45 in ^13^C-NMR and 1625–1634 cm^−1^ in the IR spectrum, respectively. The complete assignments and spectral data of compounds **8**, **13** and **23** are given in Table 1, while the structure of compounds **7** and **9** was confirmed by the single X-ray crystallographic technique (Figure 2).

### 2.2. Structure–Activity Relationships

FKB derivatives **1**–**23** were screened against two breast cancer cell lines MCF-7 (human estrogen receptor positive breast cancer cells) and MDA-MB-231 (human estrogen receptor negative) breast cancer cell lines). FKB (**1**) still possessed greater cytotoxicity (IC_50_ = 7.70 ± 0.30 and 5.90 ± 0.30 μg/mL) compared to the majority of FKB derivatives. Of the 23 tested compounds, thirteen FKB derivatives recorded higher IC_50_ values than FKB (**1**). On the other hand, FKB derivatives **2**–**4**, **7**, **9**, **13**, **15**, **16** and **22** were recorded as having potential cytotoxic effects on both MCF-7 and MDA-MB-231 cells, compared to FKB (**1**), see Table 2. Among the FKB derivatives, compounds **13**, **15** and **16** showed slightly better cytotoxicity than FKB (**1**) and flavokawain A (FKA) (**2**) for MCF-7 cells.

Among the FKB derivatives with an IC_50_ value lower than 30 μg/mL, only compound **3** and **22** showed higher sensitivity to MCF-7 cells than MDA-MB-231, indicating that the modification of the FKB skeleton helps the selectivity between these two cell lines. In short, the FKB derivatives FKB-2F (**13**), FKB-3Cl (**15**) and FKB-2Cl (**16**) exhibited greater sensitivity to both tested cell lines, warranting further evaluation of their antitumor effects. It is well established that the anticancer properties of chalcones are due to the presence of α,β-unsaturated ketone moieties. Among the tested chalcones, the 2′ hydroxyl group in ring A is essential for the cytotoxicity, as has been studied against several biological assays, predominantly its anticancer properties [38,39]. The presence of electron-withdrawing and electron-donating groups effects the α,β-unsaturated system [38,40], which eventually effects the cytotoxicity. Generally, the electron-donating group in ring A improves the cytotoxic properties, as it affects the acidity of the 2′ hydroxyl group in ring A, while the electron-donating group in ring B, as in compound **5**, **6**, **8** and **10**, did not improve the IC_50_ values at 30 μg/mL (Table 2), which possibly due to the effects of the electron-donating group on the α, β-unsaturated system. Since the α,β-unsaturated system becomes more nucleophile and the Michael receptor or protein cannot bind effectively thus activity decreases [38]. However, compounds **4** and **7** showed slightly better activity due to the selective position of the methoxy groups at **3**, **4** and **2**, **3**, which contributed less to the α,β-unsaturated ketone. In contrast, compounds with electron-withdrawing groups in ring B possessing 2Cl (**16**), 3Cl (**15**) and 2F (**13**) exhibited better cytotoxicity, see Table 2. The presence of the electron-withdrawing group in ring B, especially at position 2 and 3, as in **13** (2F), **15** (3Cl) and **16** (2Cl), contributed more to the enhancement of cytotoxicity against breast cancer cell lines (38, 39). While the presence of electron-withdrawing groups in compounds **13**, **15** and **16** pulling the electrons from the α,β-unsaturated ketone make it more electrophilic, so the receptor (nucleophile) possibly creates a strong interaction with the compound. While the electrons with the donating group, such as methoxy groups—especially at position 3, 4 or 3, 5 and 2, 4, 6, as in compounds **5**, **6**, **8**, **10**, **11**, cause rich in nucleophilicity and resonate the α,β-unsaturated carbonyl [41], eventually, the structure become less effective and binding or the interaction with the Michael receptor (nucleophile), and the compounds reduce the cytotoxic effects [38,39]. 

### 2.3. Computational Studies

The potential FKB derivatives **1**, **13**, **15** and **16** were subjected to rigid receptor docking, and the resulting poses were analyzed visually. The crystal structure of JAK2 in complex with a potent quinoxaline inhibitor, PDB: 3KRR, was downloaded in MOE and subjected to protein preparation. The results of a PASS prediction highlight the probability of the activity of these compounds as a caspase-3 stimulant, JAK2 expression inhibitor, and apoptosis agonist, as shown in Table S1. Caspase-3 belongs to the cystine–aspartic-acid protease family of proteins [42], known to play a fundamental role in the execution phase of cell death in apoptosis [43]. The activators of caspase-3, such as MX-2060 [12,44], Smac [45] and Leachianone A [46], induce apoptosis in various types of cancers. 

**Figure 3:**
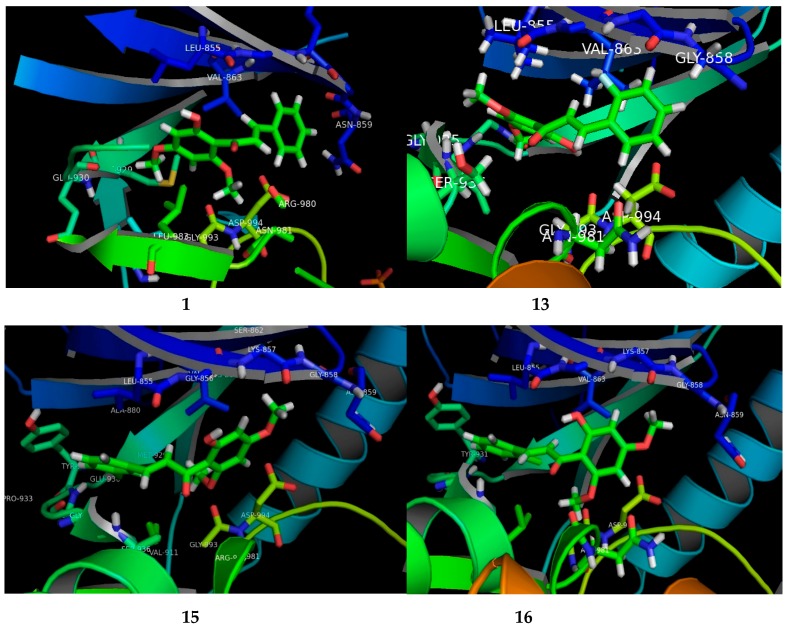
The visual presenting the interaction of FKB derivatives (**1**, **13**, **15** and **16**) with the ATP binding site of human JAK2 tyrosine kinase.

The high Pa values of the compounds (0.892–0.924) allowed us to conclude that our newly-synthesized chalcone derivatives may exhibit anticancer effects by activating the caspase-3-dependent apoptotic pathway. Moreover, the cell-specific response of the chalcone derivatives can be explained partly by the fact that the human MCF-7 breast carcinoma cells completely lack caspase-3 expression as a result of a frameshift mutation [43,47]. This loss-of-function mutation has contributed to chemotherapeutic resistance in breast cancer [12,44]. The Janus kinase-2 (JAK2) protein is targeted by many anti-cancer drugs to produce their response [45,48]. As presented in Table S1, our compounds may inhibit JAK2, with Pa values ranging from 0.821 to 0.878. We carried out a molecular docking simulation to establish the binding mode of the potent FKB derivatives with IC_50_ values in single digits, namely **1**, **13**, **15** and **16**, with JAK2 (PDB:3KRR). All the compounds fit the active site of JAK2 wells, depicted in Figure 3. The docking results revealed that all the compounds mediated hydrophobic contacts with Val863 and Leu983. The methoxy group of the compounds interacted via an acceptor–donor hydrogen bonding motif to the Leu932 backbone. A hydrogen bond with Leu932 provided these compounds with anchorage in the cavity. In the case of compound **13** and **15**, an additional hydrogen bond was observed with Leu855 and Asp944, respectively. Analysis of the average duty cycle revealed that the most stable hydrogen bond pair was Arg938 and O2, and Leu932 and O3 in the **13** and **16** complexes, respectively. The binding profile of **13**-JAK2 shows equally high expression of three different host–guest pairs, Gly856, Leu855 and Lys857. Accurate characterization of protein–ligand interactions is indispensable for the drug design. Molecular dynamic simulation is a state-of-art technique that is used to characterize the time-dependent behavior for protein–ligand complex (See Supplementary Material).

## 3. Materials and Methods

### 3.1. Chemistry

Melting points were determined on a Fisher–Johns melting apparatus and are uncorrected. UV spectra were recorded on UV-VIS spectrophotometer model of type Genesys 10s and expressed in nm. FT-IR spectroscopic studies were carried out on a FTIR spectrophotometer 1000, model Perkin Elmer, at a room temperature of 25 °C. KBr pellets were dried in an oven and scanned for calibration purposes. ^1^H-NMR spectra of compounds were recorded on a Bruker Ascend TM 600 MHz machine. The chemical shifts (*δ*) are presented with reference to CDCl_3_ (*δ*: 7.25) and tetramethylsilane (TMS) (*δ*: 0.00) as the internal reference. Electron-spray ionization mass spectra in positive mode (ESI-MS) data were recorded on a Bruker Esquire 3000+ spectrometer. Column chromatography purifications were carried out on a Silica Gel 60 (Merck, 70–230 mesh). The purity of compounds was checked by thin-layer chromatography (TLC) and ^1^H-NMR. All the chemicals were purchased from Sigma-Aldrich (Saint Louis, MO, USA). Other reagents were purchased from Sinopharm Chemical Reagent Co. Ltd., (Shanghai, China).

### 3.2. Docking Analysis Method

To rationalize the anti-cancer potential of FKB derivatives, the online web Prediction of Activity Spectra for Substances (PASS) server was used to predict the biological activity of potential FKB derivatives [49,50]. The information on the compounds was uploaded with the value of probability of activity (Pa) set to 0.7. The results were then analyzed critically to understand the molecular basis for the anti-cancer activity of the compounds. Molecular docking simulation of selected inhibitors against JAK2 was carried out using MOE [51]. The crystal structure of JAK2 in complex with a potent quinoxaline inhibitor, PDB:3KRR [52], was downloaded in MOE. The derivatives with potential IC_50_ values (Table 2.) for compound **1**, **13**, **15** and **16** were subjected to rigid receptor docking, and the resulting poses were analyzed visually. All the graphics were rendered using MOE and Chimera [53]. To study the dynamic behavior of the proposed ligand–protein complex, we have performed in a short production run of 10 ns using AMBER 14 [54]. The resulting trajectories were analyzed using the CPPTRAJ module in AMBERTOOLS 16 [55,56].

### 3.3. Synthesis of Flavokawain B derivatives

KOH (20% *w*/*v* aqueous solution) was added to a stirred solution of the appropriate acetophenone (1.0 eq) and a substituted benzaldehyde (1.25 eq.) in ethanol, and the mixture was stirred at room temperature for 48–72 h. The reaction mixture was cooled to 0 °C (ice-water bath) and acidified with HCl (10% *v*/*v* aqueous solution). In most cases, yellow precipitate was formed, filtered and washed with 10% aqueous HCl solution. In the cases where an orange-yellow layer was formed, the mixture was extracted with CH_2_Cl_2_ or ethyl acetate, the extracts was dried over Na_2_SO_4_ anhydrous and the solvent was evaporated and crystalized with methanol or ethanol.

### 3.4. Characterization Data

The data of flavokawain B (**1**) and flavokawain A (**2**) have been published previously by us [26,57].

*(E)-1-(2′-Hydroxy-4′,6′-dimethoxyphenyl)-3-(4-(methylthio)phenyl)prop-2-en-1-one* (**3**). Orange crystal (78.3% yield); m.p.: 129–131 °C; UV-Vis (CHCl_3_) λ_max_: 371–372 nm; IR (KBr, cm^−1^): 3469 (O-H), 2981–3014 (Ar C-H stretch), 1619 (C=O), 1548–1584 (C=C-C=O), 1431–1432 (Ar C=C), 1155–1218 (C-O) [36].

*(E)-3-(2,3-Dimethoxyphenyl)-1-(2′-hydroxy-4′,6′-dimethoxyphenyl)prop-2-en-1-one* (**4**). Yellow crystal (83.4% yield); m.p.: 121–123 °C (lit. [58] 120–122 °C); UV-Vis (MeOH)_λmax_: 335–337 nm; IR (KBr, cm^−1^): 3458 (O-H), 2839–2985 (Ar C-H stretch), 1627 (C=O), 1556–1581 (C=C-C=O), 1350–1442 (C=C), 1213–1268 (C-O); ^1^H-NMR (600 MHz, CDCl_3_) *δ*: 14.34 (s, 1H, OH, C-2′), 8.08 (d, 1H, H-β, *J* = 15.78 Hz), 7.96 (d, 1H, H-α, *J =* 15.78 Hz), 7.24 (d, 1H, H-6, *J =* 7.74 Hz), 7.08 (t, 1H, H-5, *J =* 8.04, 7.98 Hz), 6.95 (d, 1H, H-4, *J =* 8.04 Hz), 6.11 (d, 1H, H-3′, *J =* 2.10 Hz), 5.96 (d, 1H, H-5′, *J =* 2.10 Hz), 3.91 (s, 3H, OCH_3_, C-6′), 3.90 (s, 3H, OCH_3_, C-2), 3.89 (s, 3H, OCH_3_, C-3), 3.84 (s, 3H, OCH_3_, C-4′); ^13^C-NMR (150 MHz, CDCl_3_) *δ*: 192.93 (C=O), 168.40 (C-2), 166.18 (C-6′), 162.55 (C-4′), 153.24 (C-3), 148.88 (C-1), 137.19 (C-2′), 129.79 (C-5), 128.95 (C-6), 124.12 (C-4), 119.80 (C-5′), 113.76 (C-3′), 106.47 (C-1′), 93.70 (C-β), 91.20 (C-α), 61.31 (OCH_3_), 55.92 (OCH_3_), 55.84 (OCH_3_), 55.58 (OCH_3_); EI-MS: *m*/*z* = 344 (60), 329 (7), 313 (100), 207 (30), 181 (24), 180 (87), 164 (70), 152 (37), 149 (55), 137 (51), 121 (52), 91 (25), 77 (21), 69 (13); (Molecular formula C_19_H_20_O_6_).

*(E)-1-(2′-Hydroxy-4′,6′-dimethoxyphenyl)-3-(2,4,6-trimethoxyphenyl)prop-2-en-1-one* (**6**). Orange crystal (82.8% yield); m.p.: 158–159 °C (lit. [59] 151–153 °C); UV-Vis (MeOH): λ_max_: 386 nm; IR (KBr, cm^−1^): 3464 (O-H), 2941–3010 (Ar C-H stretch), 1618 (C=O), 1414–1545 (C=C), 1122–1217 (C-O); ^1^H-NMR (600 MHz, CDCl_3_) *δ*; 14.76 (s, 1H, OH, C-2′), 8.32 (d, 1H, H-β, *J =* 15.78 Hz), 8.25 (d, 1H, H-α, *J =* 15.78 Hz), 6.13 (s, 2H, H-3 and H-5), 6.09 (d, 1H, H-3′, *J =* 2.40 Hz), 5.94 (d, 1H, H-5′, *J =* 2.40 Hz), 3.90 (s, 3H, OCH_3_, C-6 and C-6′), 3.89 (s, 3H, OCH_3_, C-2), 3.85 (s, 3H, OCH_3_, C-4′), 3.82 (s, 3H, OCH_3_, C-4); EI-MS: *m*/*z* = 374 (2), 344 (44), 329 (8), 313 (100), 281 (10), 207 (50), 181 (25), 180 (54), 164 (44), 152 (21), 149 (37), 137 (37), 121 (33), 105 (12), 91 (26), 77 (23), 69 (15); (Molecular formula C_20_H_22_O_7_).

*(E)-3-(2,5-Dimethoxyphenyl)-1-(2′-hydroxy-4′,6′-dimethoxyphenyl)prop-2-en-1-one* (**8**). EI-MS: *m*/*z* = 344 (78), 327 (33), 282 (10), 207 (68), 191 (44), 181 (27), 164 (62), 152 (14), 151 (100), 137 (19); (Molecular formula C_19_H_20_O_6_).

*(E)-3-(2-Fluorophenyl)-1-(2′-hydroxy-4′,6′-dimethoxyphenyl)prop-2-en-1-one* (**13**). EI-MS: *m*/*z* = 302 (100), 301 (100), 285 (19), 282 (45), 207 (100), 181 (100), 166 (28), 152 (24), 149 (28), 137 (67), 121 (44), 101 (88), 95 (51), 69 (52); (Molecular formula C_17_H_15_FO_4_).

*(E)-3-(4-Fluorophenyl)-1-(2′-hydroxy-4′,6′-dimethoxyphenyl)prop-2-en-1-one* (**14**). Yellow flat crystal (80.7% yield); m.p.: 144–145 °C (lit. [17] 140–141 °C); UV-Vis (MeOH) λ_max_: 342–343 nm; IR (KBr, cm^−1^): 3460 (O-H), 2854–3118 (Ar C-H stretch), 1632 (C=O), 1573–1592 (C=C-C=O), 1345–1507 (C=C), 1115–1216 (C-O); ^1^H-NMR (600 MHz, CDCl_3_) *δ*: 14.28 (s, 1H, OH, C-2′), 7.82 (d, 1H, H-β, *J =* 15.60 Hz), 7.74 (d, 1H, H-α, *J =* 15.60 Hz), 7.59 (m, 1H, H-2), 7.58 (m, 1H, H-6), 7.10 (m, 1H, H-3), 7.09 (m, 1H, H-5), 6.11 (d, 1H, H-3′, *J =* 2.40 Hz), 5.96 (d, 1H, H-5′, *J =* 2.40 Hz), 3.92 (s, 3H, OCH_3_, C-6′), 3.84 (s, 3H, OCH_3_, C-4′); GC-MS: *m*/*z* = 302 (84), 301 (100), 285 (12), 274 (14), 207 (99), 181 (41), 153 (12), 137 (23), 121 (13), 101 (17), 69 (11); (Molecular formula C_17_H_15_FO_4_).

*(E)-3-(3-Chlorophenyl)-1-(2′-hydroxy-4′,6′-dimethoxyphenyl)prop-2-en-1-one* (**15**). Yellow solid (83.1% yield); m.p.: 105–107 °C (lit. [60] 104–106 °C); UV-Vis (MeOH) λ_max_: 338–339 nm; IR (KBr, cm^−1^): 3460 (O-H), 2945–3014 (Ar C-H stretch), 1634 (C=O), 1567–1585 (C=C-C=O), 1342–1443 (C=C), 1213–1215 (C-O); ^1^H-NMR (600 MHz, CDCl_3_) *δ*: 14.19 (s, 1H, OH, C-2′), 7.86 (d, 1H, H-β, *J =* 15.60 Hz), 7.68 (d, 1H, H-α, *J =* 15.60 Hz), 7.57 (brs, 1H, H-2), 7.46 (m, 1H, H-5) 7.35 (m, 2H, H-4 and H-6), 6.11 (d, 1H, H-3′, *J =* 2.34 Hz), 5.97 (d, 1H, H-5′, *J =* 2.34 Hz), 3.93 (s, 3H, OCH_3_, C-6′), 3.85 (s, 3H, OCH_3_, C-4′); ^13^C-NMR (150 MHz, CDCl_3_) *δ*: 192.26 (C=O), 168.47 (C-2′), 166.46 (C-4′), 162.50 (C-6′), 140.45 (C-β), 137.45 (C-1), 134.83 (C-3), 130.12 (C-2), 129.84 (C-4), 128.86 (C-5), 127.88 (C-6), 126.62 (C-α), 106.26 (C-1′), 93.81 (C-3′), 91.33 (C-5′), 55.95 (OCH_3_), 55.65 (OCH_3_); EI-MS: *m*/*z* = 317 (1), 314 (90), 297 (10), 283 (68), 267 (7), 207 (100), 181 (65), 167 (16), 152 (19), 137 (27), 121 (22); (Molecular formula C_17_H_15_ClO_4_).

*(E)-3-(2-Chlorophenyl)-1-(2′-hydroxy-4′,6′-dimethoxyphenyl)prop-2-en-1-one* (**16**). Yellow crystal (80.7% yield); m.p.: 132–134 °C (lit. [18] 135–136 °C); UV-Vis (MeOH) λ_max_: 341–342 nm; IR (KBr, cm^−1^): 3448 (O-H), 2957–3132 (Ar C-H stetch), 1632 (C=O), 1556–1585 (C=C-C=O), 1338–1435 (C=C), 1110–1219 (C-O), 745–748 (C-Cl); ^1^H-NMR (600 MHz, CDCl_3_) *δ*: 14.24 (s, 1H, OH, C-2′), 8.12 (d, 1H, H-β, *J =* 15.60 Hz), 7.85 (d, 1H, H-α, *J =* 15.60 Hz), 7.67 (m, 1H, H-6), 7.40 (m, 1H, H-3), 7.28 (m, 2H, H-4 and H-5), 6.08 (d, 1H, H-3′, *J =* 2.34 Hz), 5.93 (d, 1H, H-5′, *J =* 2.40 Hz), 3.88 (s, 3H, OCH_3_, C-6′), 3.81 (s, 3H, OCH_3_, C-4′); EI-MS: *m*/*z* = 318 (30), 301 (4), 283 (70), 267 (7), 207 (100), 181 (32), 165 (6), 152 (6), 137 (12), 101 (12), 69 (7); (Molecular formula C_17_H_15_ClO_4_).

*(E)-3-(4-Chlorophenyl)-1-(2′-hydroxy-4′,6′-dimethoxyphenyl)prop-2-en-1-one* (**17**). Yellow crystal (84.3% yield); m.p.: 171–173 °C (lit. [61] 173–175 °C); UV-Vis (MeOH) λ_max_: 342–343 nm; IR (KBr, cm^−1^): 3420 (O-H), 2981–3018 (Ar C-H stretch), 1630 (C=O), 1488–1591 (C=C), 1217–1219 (C-O), 820–822 (C-Cl); ^1^H-NMR (600 MHz, CDCl_3_) *δ*: 14.24 (s, 1H, OH, C-2′), 7.85 (d, 1H, H-β, *J =* 15.60 Hz), 7.72 (d, 1H, H-α, *J =* 15.60 Hz), 7.53 (d, 1H, H-2, *J =* 8.46 Hz), 7.52 (d, 1H, H-6, *J =* 8.46 Hz), 7.38 (d, 1H, H-3, *J =* 8.46 Hz), 7.37 (d, 1H, H-5, *J =* 8.46 Hz), 6.11 (d, 1H, H-3′, *J =* 2.40 Hz), 5.96 (d, 1H, H-5′, *J =* 2.40 Hz), 3.92 (s, 3H, OCH_3_, C-6′), 3.84 (s, 3H, OCH_3_, C-4′); EI-MS: *m*/*z* = 318 (57), 301 (7), 283 (2), 207 (100), 181 (34), 165 (13), 152 (19), 137 (25), 111 (6), 102 (20); (Molecular formula C_17_H_15_ClO_4_).

*(E)-3-(4-Bromophenyl)-1-(2′-hydroxy-4′,6′-dimethoxyphenyl)prop-2-en-1-one* (**18**). Yellow flat crystal (80.5% yield); m.p.: 165.8–167.5 °C (lit. [17] 150–151 °C); UV-Vis (MeOH) λ_max_: 346 nm; IR (KBr, cm^−1^): 3447 (O-H), 2949–3010 (Ar C-H stretch), 1633 (C=O), 1568–1590 (C=C-C=O), 1338–1488 (C=C), 1215–1218 (C-O); ^1^H-NMR (500 MHz, CDCl_3_) *δ*: 7.85 (d, 1H, H-β, *J =* 15.50 Hz), 7.68 (d, 1H, H-α, *J =* 15.50 Hz), 7.53 (d, 1H, H-2, *J =* 8.50 Hz), 7.51 (d, 1H, H-6, *J =* 8.50 Hz), 7.45 (d, 1H, H-3, *J =* 8.50 Hz), 7.43 (d, 1H, H-5, *J =* 8.50 Hz), 6.09 (d, 1H, H-3′, *J =* 2.00 Hz), 5.95 (d, 1H, H-5′, *J =* 2.50 Hz), 3.90 (s, 3H, OCH_3_, C-6′), 3.83 (s, 3H, OCH_3_, C-4′); ^13^C-NMR (150 MHz, CDCl_3_) *δ*: 192.31 (C=O), 168.45 (C-2′), 166.40 (C-4′), 162.48 (C-6′), 140.78 (C-β), 134.53 (C-1), 132.10 (C-3), 132.10 (C-5), 129.68 (C-2), 129.68 (C-6), 128.15 (C-α), 124.19 (C-4), 106.29 (C-1′), 93.85 (C-3′), 91.32 (C-5′), 55.89 (OCH_3_), 55.61 (OCH_3_); EI-MS: *m*/*z* = 363 (48), 362 (48), 346 (5), 281 (5), 209 (16), 207 (100), 181 (25), 152 (9); (Molecular formula C_17_H_15_BrO_4_).

*(E)-1-(2′-Hydroxy-4′,6′-dimethoxyphenyl)-3-(3-nitrophenyl)prop-2-en-1-one* (**20**). Yellow cotton (82.6% yield); m.p.: 169–171 °C (lit. [61] 169–170 °C); UV-Vis (MeOH) λ_max_: 336 nm; IR (KBr, cm^−1^): 3460 (O-H), 2851–3092 (Ar C-H stretch), 1638 (C=O), 1583 (C=C-C=O), 1427–1525 (C=C), 1223–1225 (C-O); ^1^H-NMR (500 MHz, CDCl_3_) *δ*: 8.56 (brs, 1H, H-2), 8.30 (d, 1H, H-4, *J =* 8.20 Hz), 8.22 (d, 1H, H-6, *J =* 7.75 Hz), 8.15 (d, 1H, H-β, *J =* 15.70 Hz), 7.84 (d, 1H, H-α, *J =* 15.70 Hz), 7.78 (t, 1H, H-5, *J =* 7.95, 8.00 Hz), 6.17 (d, 1H, H-3′, *J =* 2.35 Hz), 6.14 (d, 1H, H-5′, *J =* 2.35 Hz), 4.04 (s, 3H, OCH_3_, C-6′), 3.91 (s, 3H, OCH_3_, C-4′); EI-MS: *m*/*z* = 329 (33), 301 (5), 281 (6), 254 (5), 207 (100), 181 (25), 152 (5), 95 (5), 73 (8); (Molecular formula C_17_H_15_NO_6_).

*(E)-3-(5-Bromo-2-hydroxyphenyl)-1-(2′-hydroxy-4′,6′-dimethoxyphenyl)prop-2-en-1-one* (**22**). Yellow crystal (76.2% yield); m.p.: 166–168 °C (lit. [61] 177–178 °C); UV-Vis (MeOH) λ_max_: 359 nm; IR (KBr, cm^−1^): 3435 (O-H), 2945–2994 (Ar C-H stretch), 1623 (C=O), 1419–1590 (C=C), 1220–1221 (C-O); ^1^H-NMR (600 MHz, CDCl_3_) *δ*: 14.19 (s, 1H, OH, C-2′), 8.12 (d, 1H, H-β, *J =* 15.70 Hz), 8.01 (d, 1H, H-α, *J =* 15.70 Hz), 7.78 (brs, 1H, H-6), 7.39 (d, 1H, H-4, *J =* 8.70 Hz), 6.97 (d, 1H, H-3, *J =* 8.64 Hz), 6.13 (brs, 1H, H-3′), 6.12 (brs, 1H, H-5′), 3.98 (s, 3H, OCH_3_, C-6′), 3.88 (s, 3H, OCH_3_, C-4′); EI-MS: *m*/*z* = 379 (1), 364 (14), 333 (5), 305 (3), 281 (9), 251 (3), 227 (9), 214 (17), 207 (60), 191 (10), 142 (13), 115 (8), 95 (10), 73 (14); (Molecular formula C_17_H_15_BrO_5_).

*(E)-3-(2-Bromo-3-hydroxy-4-methoxyphenyl)-1-(2′-hydroxy-4′,6′-dimethoxyphenyl)prop-2-en-1-one* (**23**). Orange crystal (85.6% yield); m.p.: 201–203 °C; UV-Vis (MeOH) λ_max_: 366–369 nm; IR (KBr, cm^−1^): 3425 (O-H), 2838–3014 (Ar C-H stretch), 1634 (C=O), 1496–1596 (C=C), 1213–1259 (C-O); EI-MS: *m*/*z* = 409 (4), 408 (4), 403 (3), 229 (3), 207 (3), 202 (3), 181 (2), 165 (3), 141 (4), 112 (5), 87 (4), 59 (4); (Molecular formula C_18_H_17_BrO_6_).

### 3.5. X-ray Crystallographic Analysis

X-ray analysis for all these samples was performed using Bruker APEX II DUO CCD diffractometer, employing MoKα radiation (λ = 0.71073 Å) with φ and ω scans, at room temperature. Data reduction and absorption correction were performed using the SAINT and SADABS programs [62]. All structures were solved by direct methods and refined by full-matrix, least-squares techniques on *F*^2^ using the SHELXTL software package [63]. Crystallographic data for the reported structures have been deposited at the Cambridge Crystallographic Data Centre (CCDC) with the CCDC deposition numbers of 1548733 and 1548734. Copies of available material can be obtained free of charge on application to the CCDC, 12 Union Road, Cambridge CB2 1EZ, UK, (Fax: +44-(0)1223–336033 or e-mail: deposit@ccdc.cam.ac.uk). The ORTEP (Oak Ride Thermal Ellipsoid Plot Program) of compound **7** and **9** are shown in Figure 2 and X-ray crystallographic data presented in Table 3.

### 3.6. Anticancer Activity

#### 3.6.1. Sample preparation

Stock samples at 1 mg/mL of dimethyl sulfoxide (DMSO) (Sigma-Aldrich) were prepared and kept at 4 °C.

#### 3.6.2. MTT Cell Viability Assay

Breast cancer MCF-7 and MDA-MB-231 cells were purchased from the American Type Culture Collection (ATCC, Manassas, VA, USA) and cultured at 37 °C, 5% CO_2_ and 90% humidity using RPMI-1640 medium (Sigma-Aldrich) supplemented with 10% fetal bovine serum (FBS) (Thermo Fisher Scientific, USA). The MTT [3-(4,5-dimethylthiazol-2-yl)-2,5-diphenyltetrazolium bromide] method cell viability assay was conducted according to our previous report [64]. In brief, MCF-7 and MDA-MB-231 were seeded overnight in 96-well plates at 8 × 10^4^ cells/well. All compounds were dissolved in dimethyl sulfoxide (DMSO, Sigma, St. Louis, MO, USA) to get a stock solution of 1 mg/mL and stored at 4 °C. Then, the compounds were serially diluted into the seeded cells at concentrations ranging between 30–0.47 µg/mL. Cells treated with 3% DMSO (Sigma) were used as a negative control. After 72 h of incubation, 20 µL of MTT solution (5 mg/mL) was added into all the wells and the plates were further incubated for another 3 hrs. Then, 170 µL of solution from all the wells were discarded and 100 µL of DMSO (Sigma) were added to dissolve the purple crystals. The absorbance was then recorded using an ELISA plate reader (Biotek Instruments, Winooski, VT, USA) at the wavelength of 570 nm. All samples were tested for triplicates. The percentage of cell viability was calculated using following formula:

Cell viability (%) = [OD sample at 570 nm/OD negative control at 570 nm] × 100%(1)

The IC_50_ value (concentration of compounds inhibited 50% of cell viability) was determined from the graph of cell viability vs absorbance.

## 4. Conclusions

Twenty-three FKB derivatives were synthesized by Claisen–Schmidt condensation reaction. Three FKB derivatives, namely **8**, **13** and **23**, were found to be new chalcones. The chalcones with electron-withdrawing and electron-donating substituents had effects on cytotoxicity. The FKB derivatives with electron-withdrawing groups in ring B, as in **13** (2F), **15** (3Cl) and **16** (2Cl), exhibited potential cytotoxic effects on breast cancer cell lines, while the presence of the substituted electron-donating groups, as in **4**, **5**, **9** and **10**, showed lower cytotoxic effects on MCF-7 and MDA-MB-231 cell lines. The FKB derivatives **16** (IC_50_ = 6.50 ± 0.40 and 4.12 ± 0.20 μg/mL), **15** (IC_50_ = 5.52 ± 0.35 and 6.50 ± 1.40 μg/mL) and **13** (IC_50_ = 7.12 ± 0.80 and 4.04 ± 0.30 μg/mL) were found to be the most active compounds among the FKB series chalcones. The structure–activity relationship showed FKB chalcones with different substituents and effects on cytotoxicity. The FKB skeleton is unique for anticancer agents, additionally, the presence of halogens (Cl and F) in position two and three also improve the cytotoxicity of FKB (**1**). These findings could help to improve or find new drugs for future anti-cancer agents.

## Figures and Tables

**Table 1:** ^1^H- (600 MHz) and ^13^C- (150 MHz) NMR data for compound **8**, **13** and **23**.

Carbon		8			13′			23	
	^1^H (*δ*)	Multiplicity	^13^C (*δ*)	^1^H (*δ*)	Multiplicity	^13^C (*δ*)	^1^H (*δ*)	Multiplicity	^13^C (*δ*)
1′	-	-	106.89	-	-	106.38	-	-	106.25
2′	-	-	162.36	-	-	162.57	-	-	161.84
3′	6.16	(d, *J* = 2.22 Hz, 1H)	94.38	6.11	(d, *J* = 2.40 Hz, 1H)	93.81	6.00	(d, *J* = 2.50 Hz, 1H)	93.86
4′	-	-	165.98	-	-	168.42	-	-	165.57
5′	6.13	(d, *J* = 2.46 Hz, 1H)	91.59	5.96	(d, *J* = 2.34 Hz, 1H)	91.29	5.88	(d, *J* = 2.35 Hz, 1H)	91.06
6′	-	-	165.98	-	-	166.37	-	-	165.55
1	-	-	124.18	-	-	124.40	-	-	127.05
2	-	-	153.65	-	-	134.87	-	-	113.28
3	7.06	(d, *J* = 9.06 Hz, 1H)	113.65	7.11	(m, 1H)	116.29	-	-	140.87
4	7.03	(d, *J* = 9.06 Hz, 1H)	113.58	7.18	(td, *J* = 7.56 Hz, 7.50 Hz, 1H)	131.28	-	-	149.86
5	-	-	153.16	7.59	(td, *J* = 7.56 Hz, 7.68 Hz, 1H)	129.73	6.78	(d, *J* = 8.55 Hz, 1H)	110.95
6	7.22	(brs, *J* = 2.76 Hz, 1H)	118.10	7.35	(m, 1H)	130.27	7.20	(d, *J* = 8.60 Hz, 1H)	118.85
α	7.84	(d, *J* = 15.80 Hz, 1H, H-α)	128.59	7.86	(d, *J* = 15.78 Hz, 1H, H-α)	123.73	7.67	(d, *J* = 15.45 Hz, 1H, H-α)	127.05
β	7.86	(d, *J* = 15.80 Hz, 1H, H-β)	137.83	8.00	(d, *J* = 15.78 Hz, 1H, H-β)	160.83	8.02	(d, *J* = 15.45 Hz, 1H, H-β)	144.09
OCH_3_ (C4′)	3.84	(s, 3H)	56.62	3.84	(s, 3H)	55.78	3.75	(s, 3H)	56.15
OCH_3_ (C6′)	3.89	(s, 3H)	56.58	3.91	(s, 3H)	55.60	3.85	(s, 3H)	55.63
OCH_3_ (C2)	3.82	(s, 3H)	55.95	-	-	-	-	-	-
OCH_3_ (C4)	-	-	-	-	-	-	3.81	(s, 3H)	56.22
OCH_3_ (C5)	3.77	(s, 3H)	56.16	-	-	-	-	-	-
OH (C2′)	13.46	(s, 1H)	-	14.25	(s, 1H)	-	-	(s, 1H)	-
C=O	-	-	193.03	-	-	192.62	-	-	191.86

**Table 2:** IC_50_ values (μg/mL) of FKB derivatives after 72 h treatment.

Compounds Substituted Groups						IC_50_ Values (μg/mL)	
Compounds	R1	R2	R3	R4	R5	MCF-7	MDA-MB-231
**1**	H	H	H	H	H	7.70 ± 0.30	5.90 ± 0.30
**2**	H	H	CH_3_O	H	H	8.90 ± 0.60	6.80 ± 0.45
**3**	H	H	SCH_3_	H	H	12.30 ± 1.40	18.10 ± 1.10
**4**	CH_3_O	CH_3_O	H	H	H	25.00 ± 1.80	21.10 ± 1.20
**5**	CH_3_O	H	CH_3_O	H	H	>30	>30
**6**	CH_3_O	H	CH_3_O	H	CH_3_O	>30	>30
**7**	H	CH_3_O	CH_3_O	H	H	9.70 ± 0.70	8.30 ± 0.56
**8**	CH_3_O	H	H	CH_3_O	H	>30	>30
**9**	H	CH_3_O	H	H	H	8.43 ± 0.40	7.22 ± 0.70
**10**	H	CH_3_O	H	CH_3_O	H	>30	>30
**11**	H	H	CH_3_	H	H	>30	>30
**12**	CH_3_O	H	H	H	H	>30	9.50 ± 0.60
**13**	F	H	H	H	H	7.12 ± 0.80	4.04 ± 0.30
**14**	H	H	F	H	H	>30	>30
**15**	H	Cl	H	H	H	5.50 ± 0.35	5.50 ± 0.40
**16**	Cl	H	H	H	H	6.50 ± 0.40	4.12 ± 0.20
**17**	H	H	Cl	H	H	>30	>30
**18**	H	H	Br	H	H	>30	>30
**19**	H	CH_3_O	OH	H	H	>30	27.00 ± 1.50
**20**	H	NO_2_	H	H	H	13.30 ± 3.10	17.10 ± 2.15
**21**	H	H	N(CH_3_)_2_	H	H	>30	20.5 ± 1.60
**22**	OH	H	H	Br	H	6.50 ± 0.35	14.16 ± 1.10
**23**	Br	OH	OCH_3_	H	H	>30	>30

**Table 3:** Crystal data and parameters for the structure refinement of **7** and **9**.

Compound	7	9
CCDC Number	1548734	1548733
Molecular Formula	C_19_H_20_O_6_	C_18_H_18_O_5_
Molecular Weight	344.35	314.32
Crystal System	Triclinic	Orthorhombic
Space Group	*P*ī	*Pbca*
a (Å)	8.4560 (17)	14.447 (3)
b (Å)	8.4790 (17)	7.9755 (15)
c (Å)	12.549 (3)	26.203 (5)
α (°)	104.166 (3)	90
β (°)	92.063 (3)	90
γ (°)	106.227 (3)	90
*V* (Å^3^)	832.4 (3)	3019.0 (10)
*Z*	2	8
*D*calc (g cm^−3^)	1.374	1.383
Crystal dimensions (mm)	0.58 × 0.18 × 0.11	0.55 × 0.27 × 0.14
μ (mm^−1^)	0.10	0.10
*T*_min_/*T*_max_	0.7665, 0.9584	0.8489, 0.9495
Reflections measured	28,367	14,974
Ranges/indices (*h*, *k*, *l*)	−11→11; −11→11; −17→17	−16→16; −9→9; −30→28
θ limit (°)	1.7−29.2	1.6−24.6
Unique reflections	4491	2516
Observed reflections (*I* > 2σ(*I*))	2352	1594
Parameters	234	211
Goodness of fit on *F*^2^	1.02	1.05
*R*_1_, *wR*_2_ [*I* ≥ 2σ(*I*)]	0.055, 0.176	0.056, 0.156

## Supplementary Materials

The following docking study is available online, Figure S1: the graph presenting the trend of root mean square deviation (Ǻ) of all the four systems in the present study, Figure S2 and Table S1: the difference in the initial and final coordinates of FKB1-JAK2 complex. The cyan color illustrates the initial pose (1 ns) while the final pose (10 ns) is presented in magenta. The ligand presented significant deviation from the initial poses, which explains the abrupt hydrogen bonding profile of the system, Table S1: the selected biological activities and their probabilities of activity (Pa) and (Pi) as obtained from the PASS online server.
