# Supplementary material for: Design, Synthesis and Docking Studies of Flavokawain B Type Chalcones and Their Cytotoxic Effects on MCF-7 and MDA-MB-231 Cell Lines
Source: Molecules. 2018 Mar 8;23(3):616. doi: 10.3390/molecules23030616 (PMC6017189; doi:10.3390/molecules23030616)
Supplement: Supplementary file 1 [file molecules-23-00616-s001.zip › IR spectra (supplementary data molecules paper).pdf]

1. FKB (**1**)

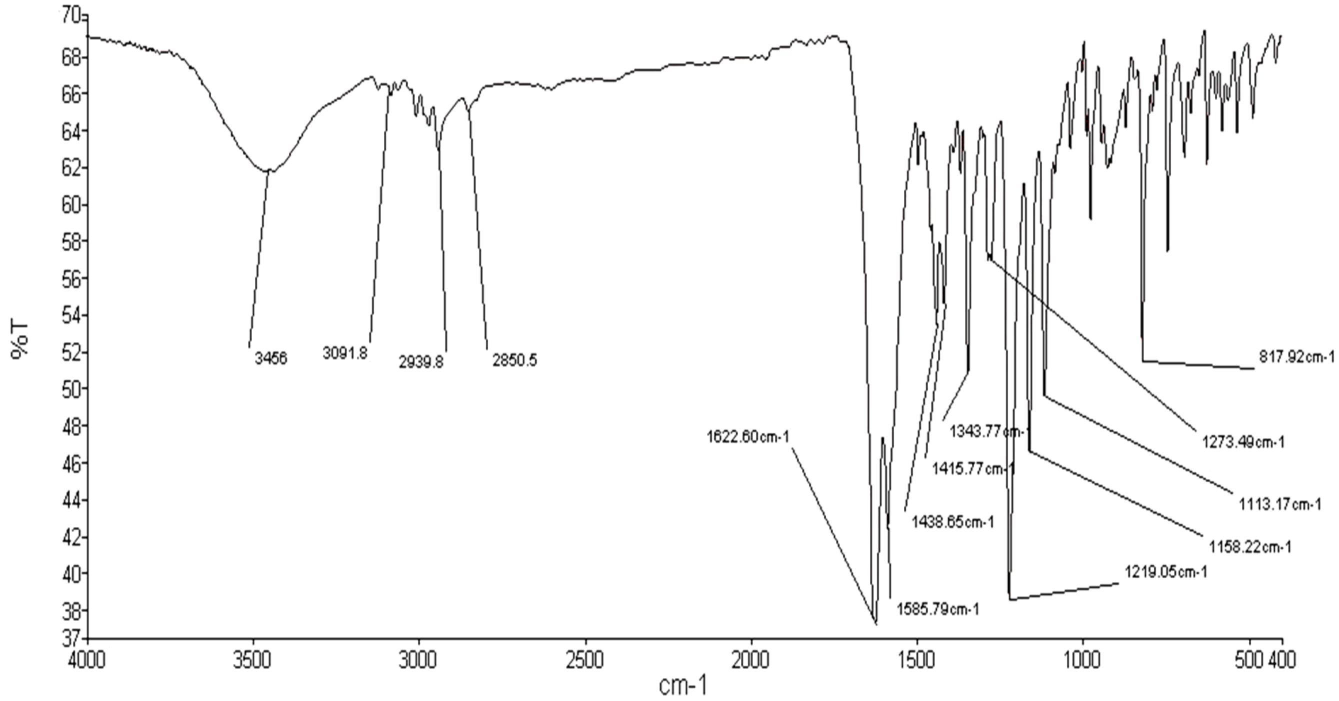
%T
cm-1
3456
3091.8
2939.8
2850.5
1622.60cm-1
1585.79cm-1
1438.65cm-1
1415.77cm-1
1343.77cm-1
1219.05cm-1
1158.22cm-1
1113.17cm-1
1273.49cm-1
817.92cm-1

2. FKA (**2**)

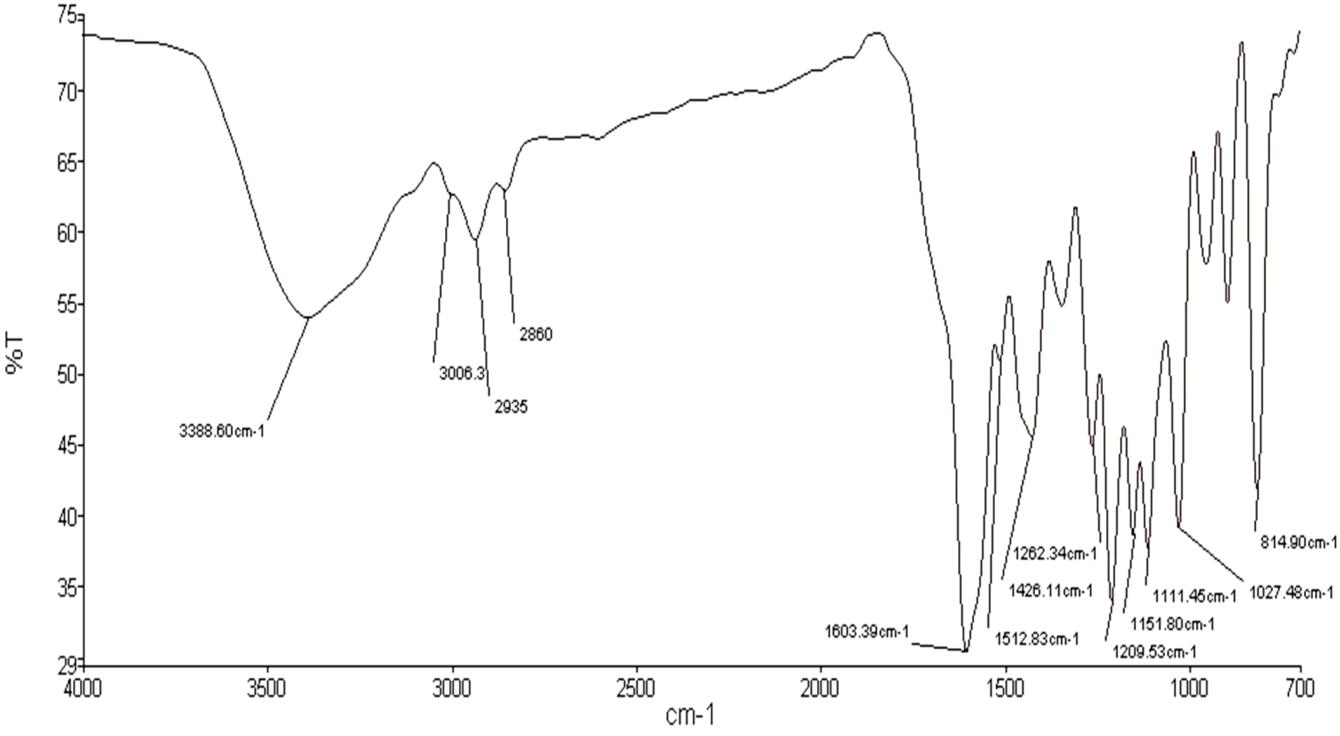
%T
cm-1
3388.60cm-1
3006.3
2935
2860
1603.39cm-1
1512.83cm-1
1426.11cm-1
1262.34cm-1
1209.53cm-1
1151.80cm-1
1111.45cm-1
1027.48cm-1
814.90cm-1

3. FKS (**3**)

1.

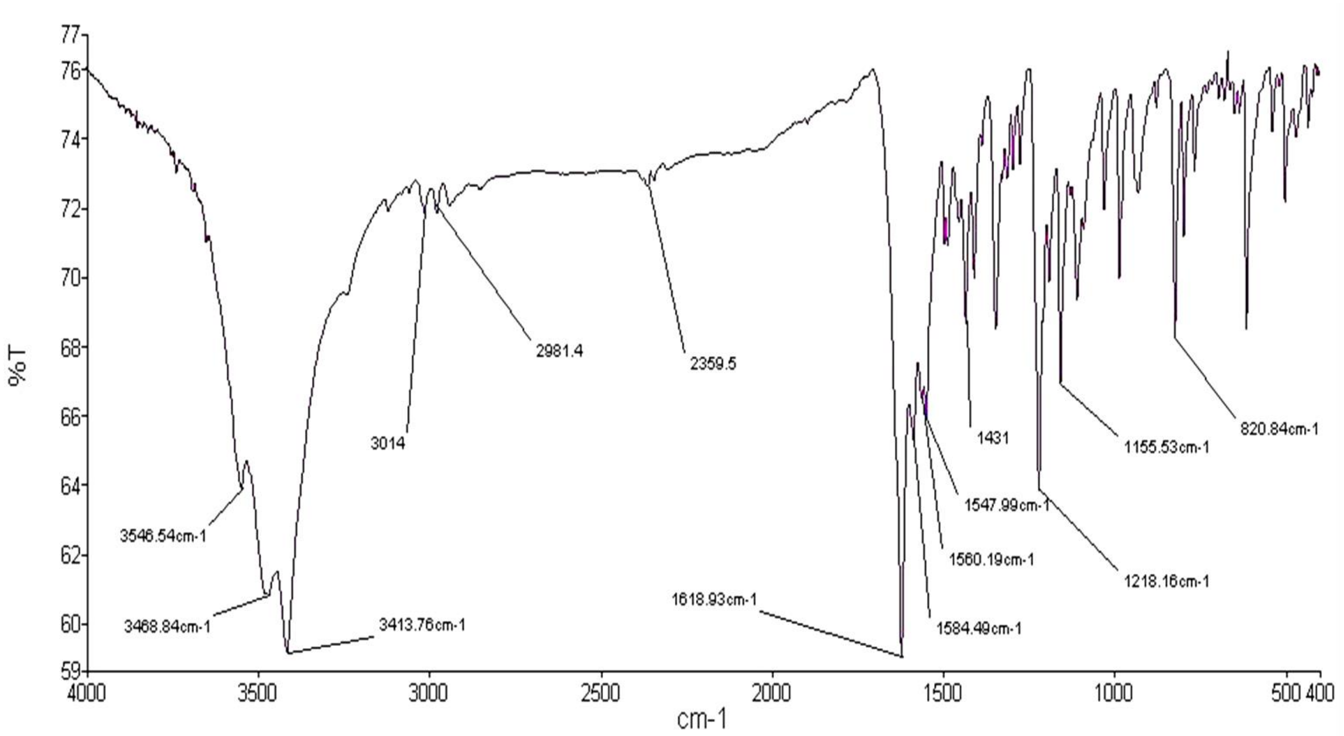

4.Fk2,3 dm (**4**)

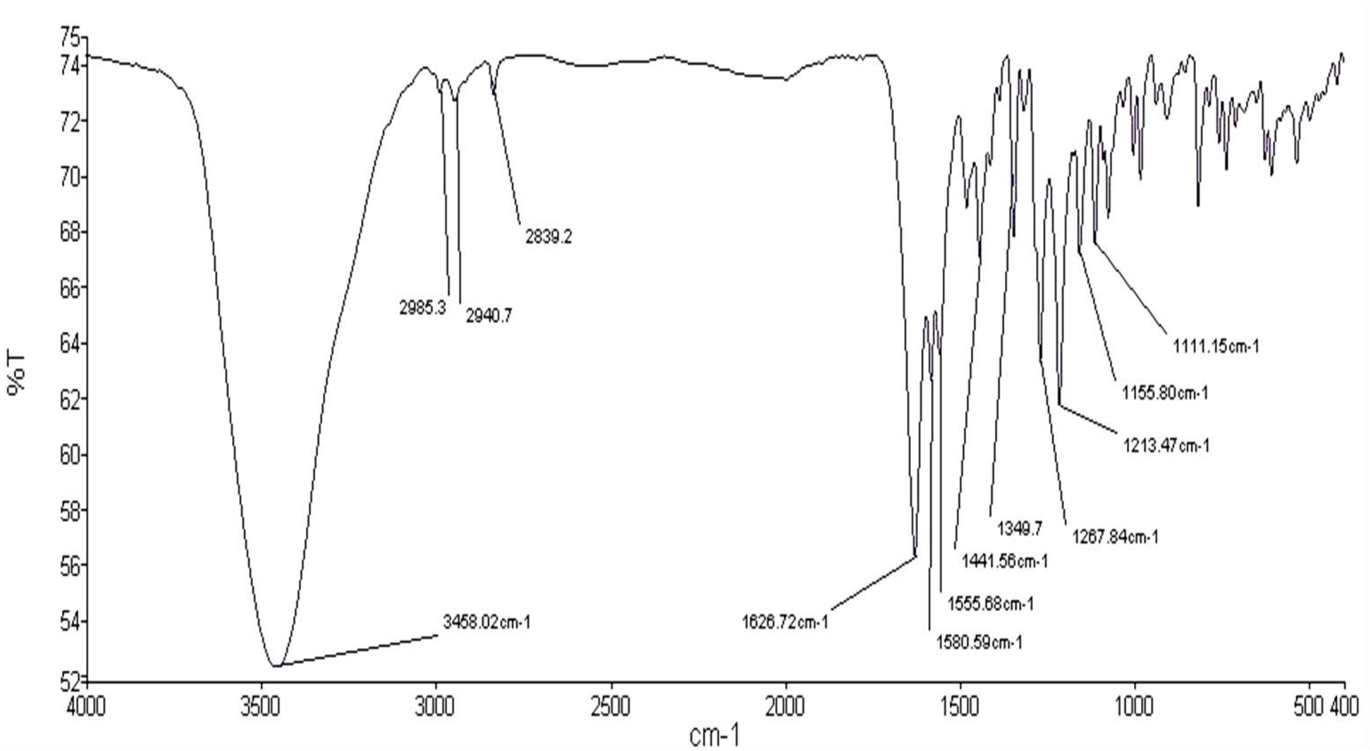

5.Fk2,4 dm (5)

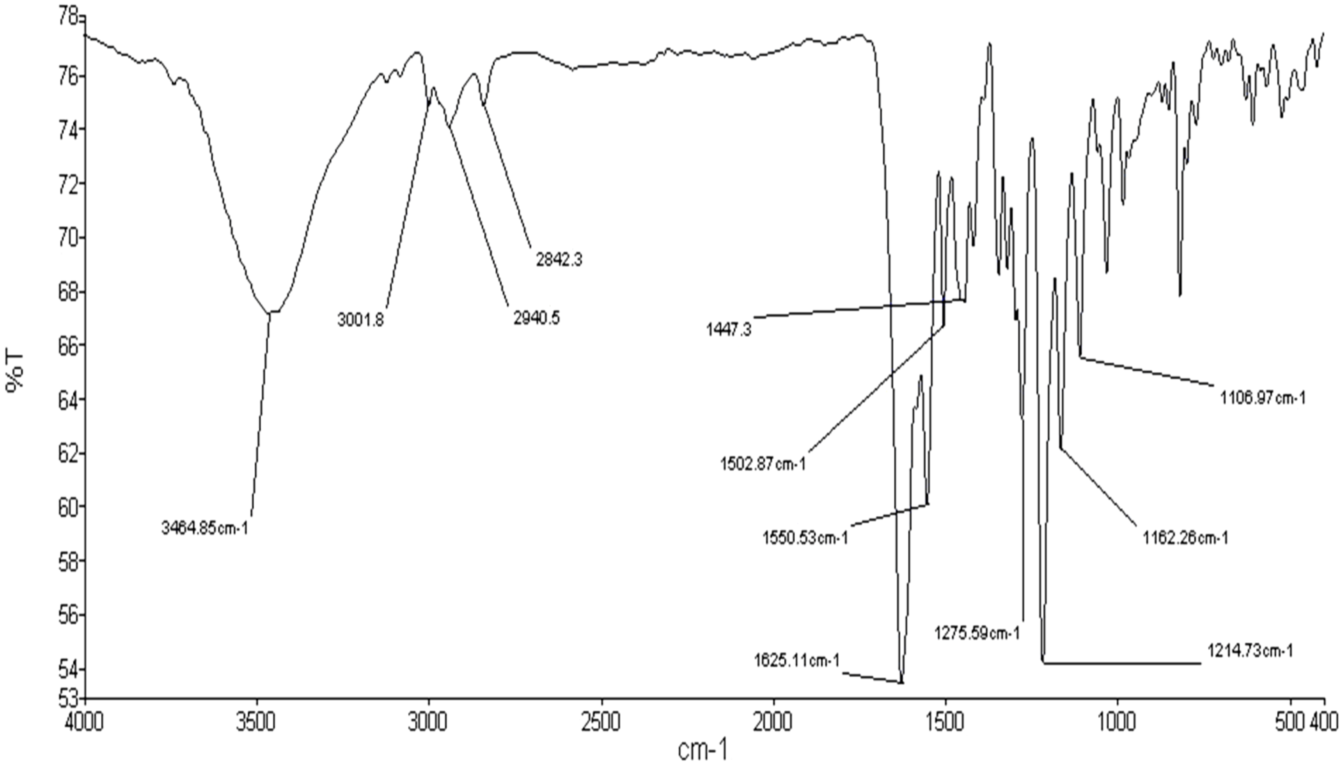

6. Fk2,4,6 (**6**)

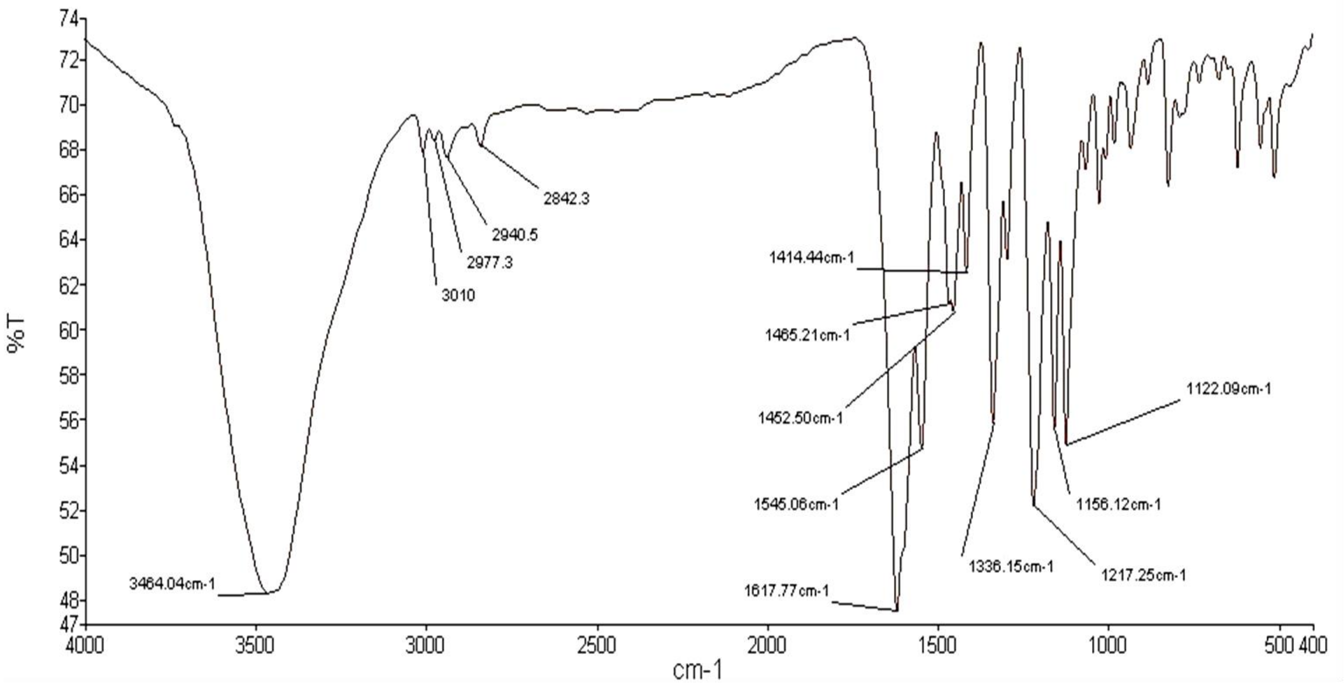

7. Fk3,4 dm (**7)**

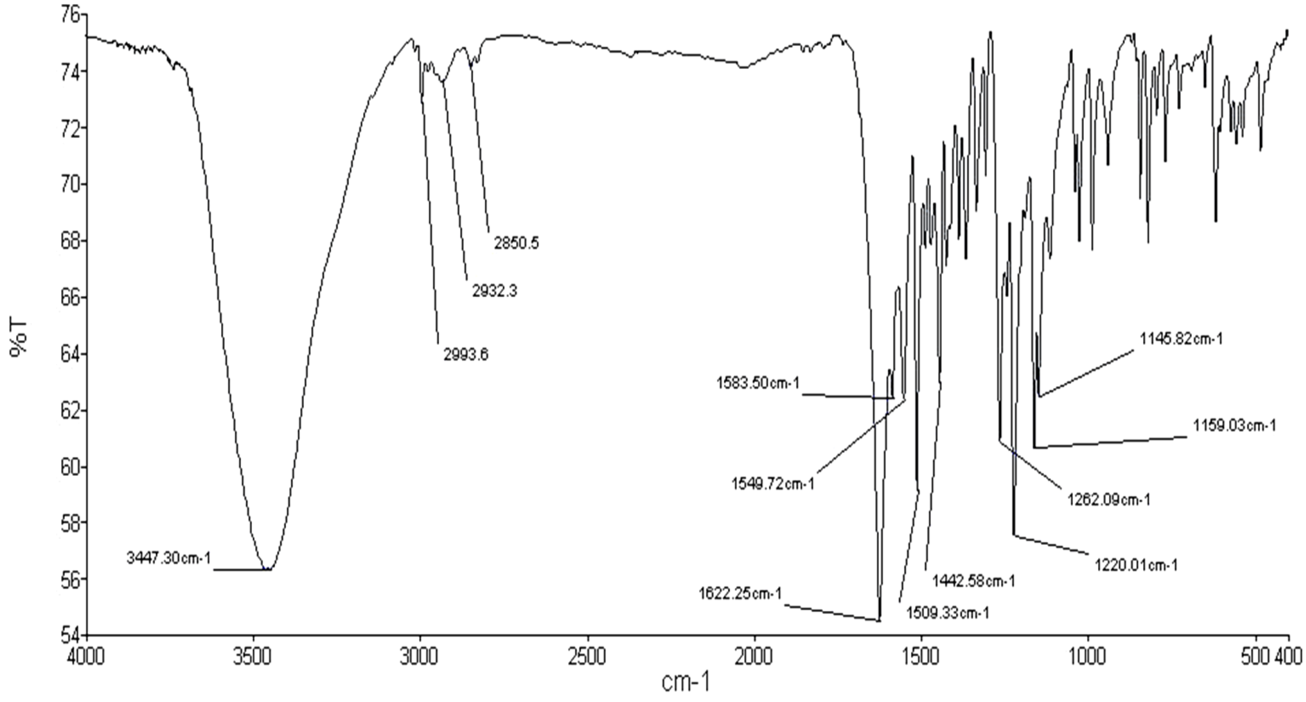

8. Fk2,5 dm (**8**)

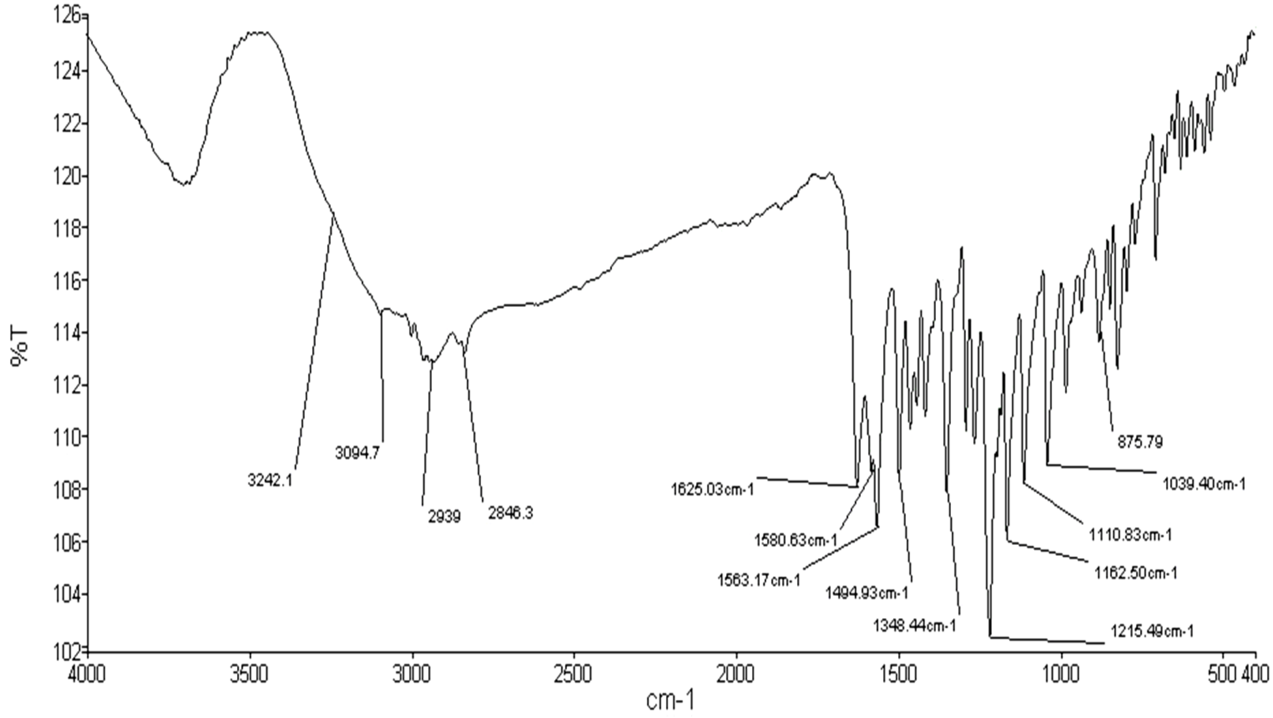

9. Fk3m (**9**)

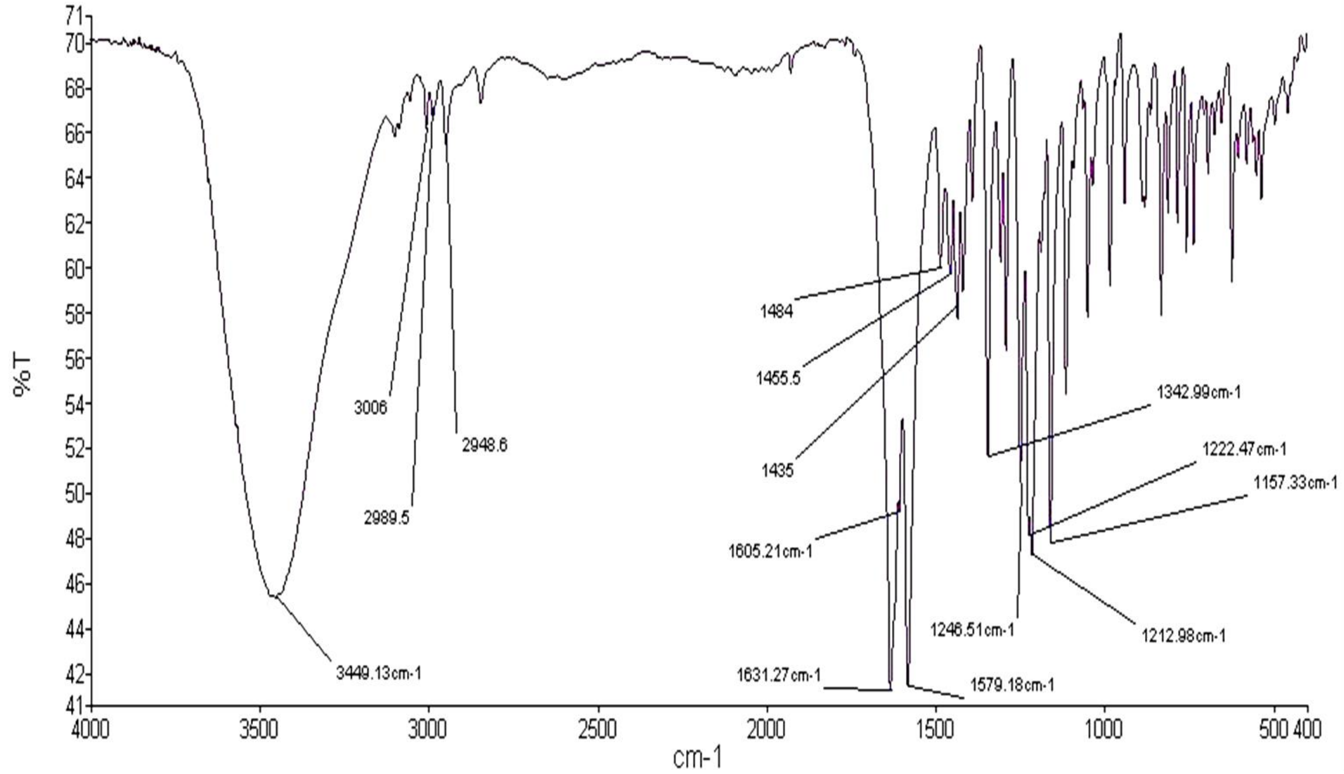

10. Fk3,5 dm (**10**)

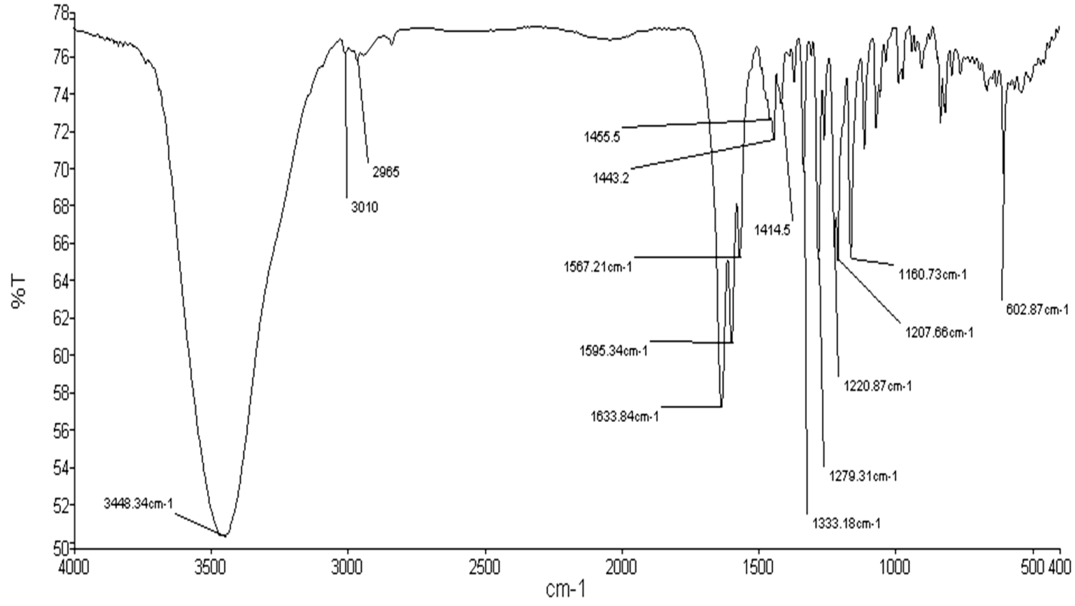

11. Fk4m (**11**)

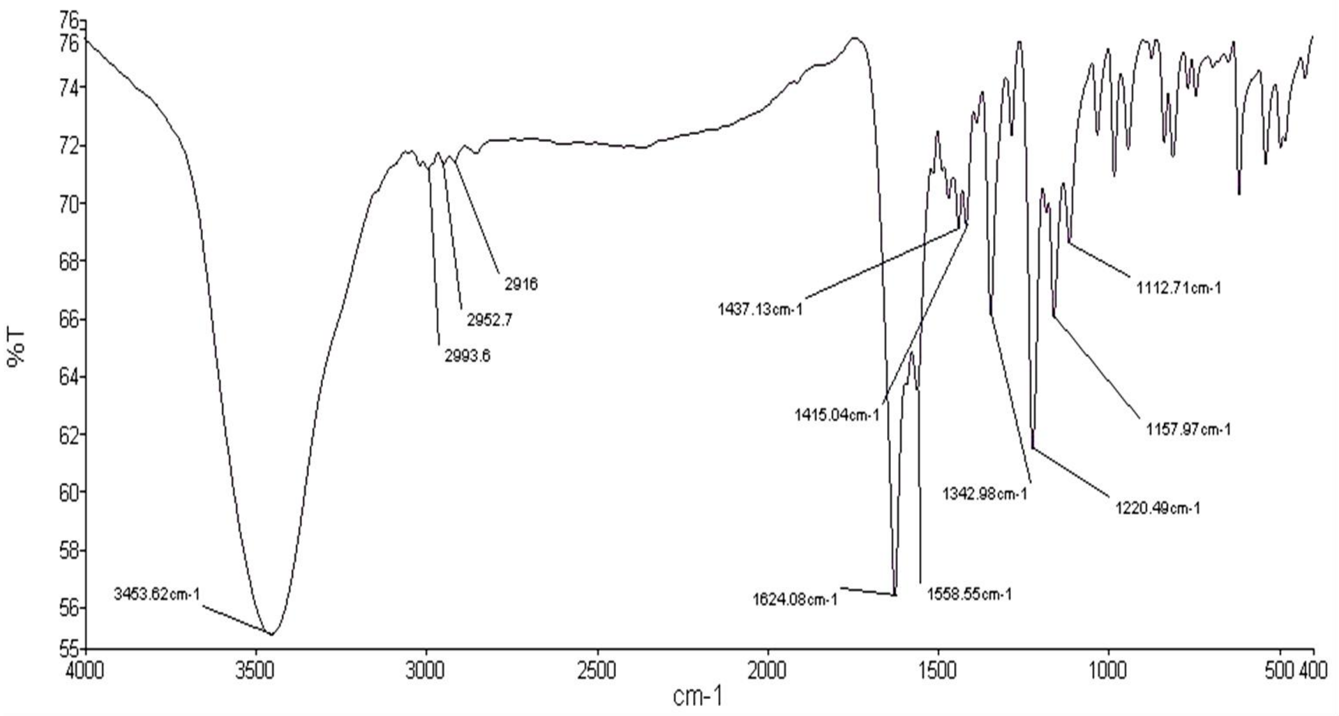

12. Fk2m (**12**)

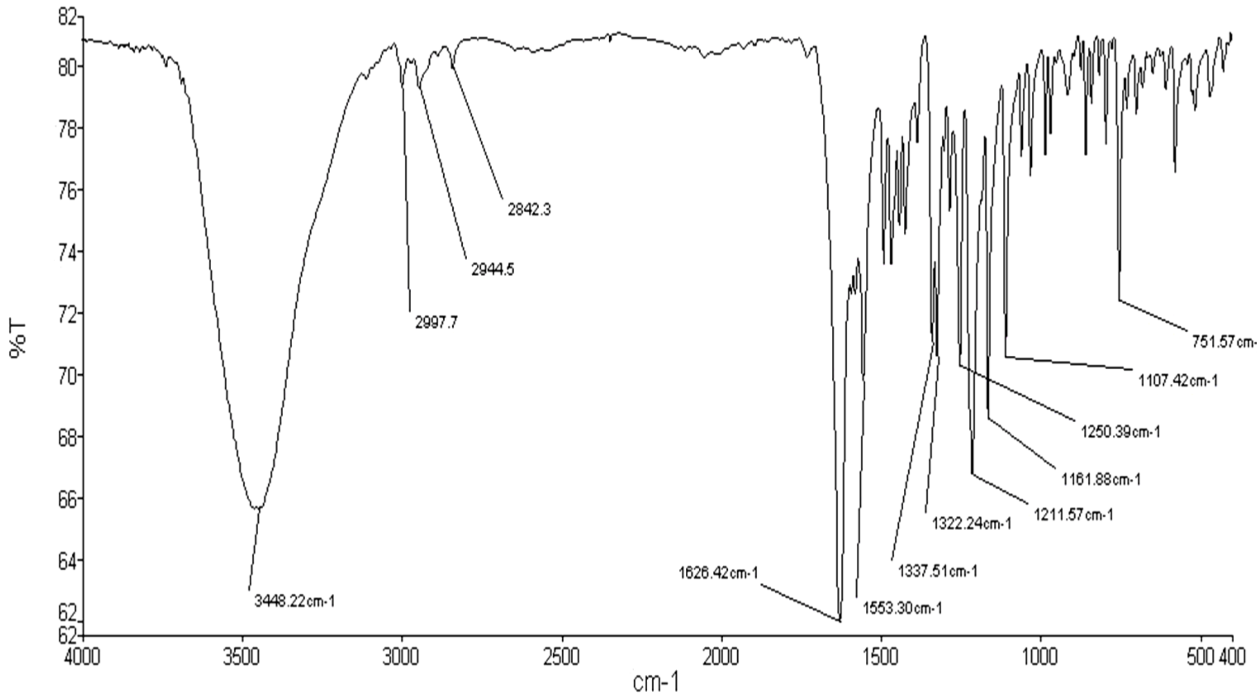

13. Fk2F (**13**)

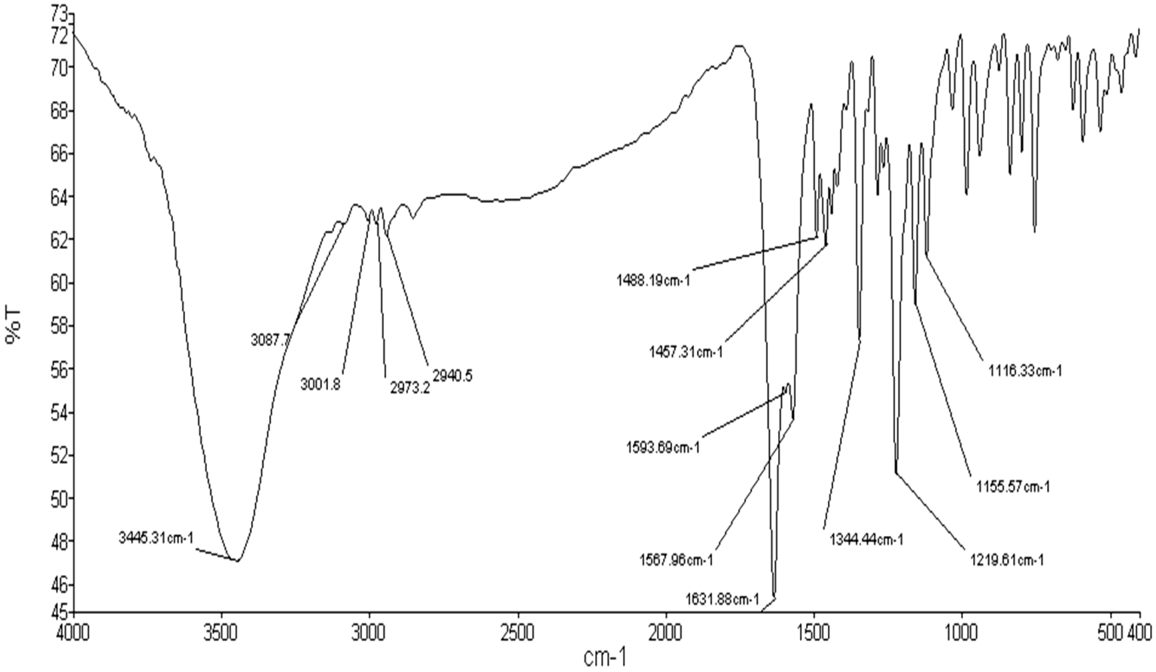

14. Fk4F (**14**)

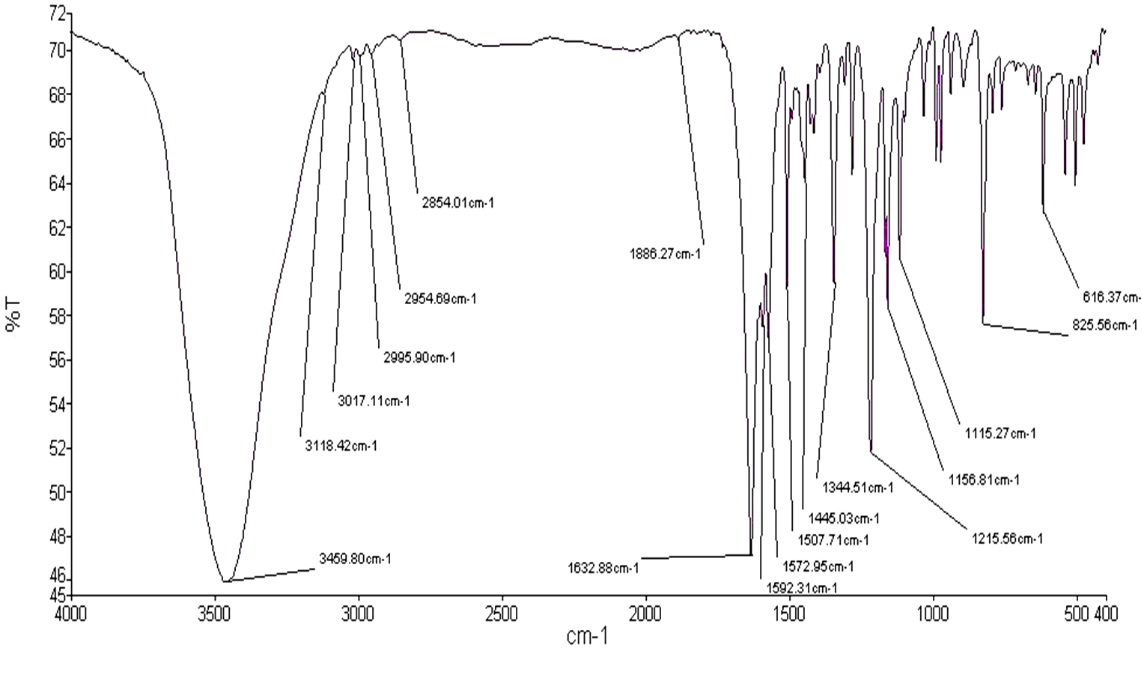

15. Fk3Cl (**15**)

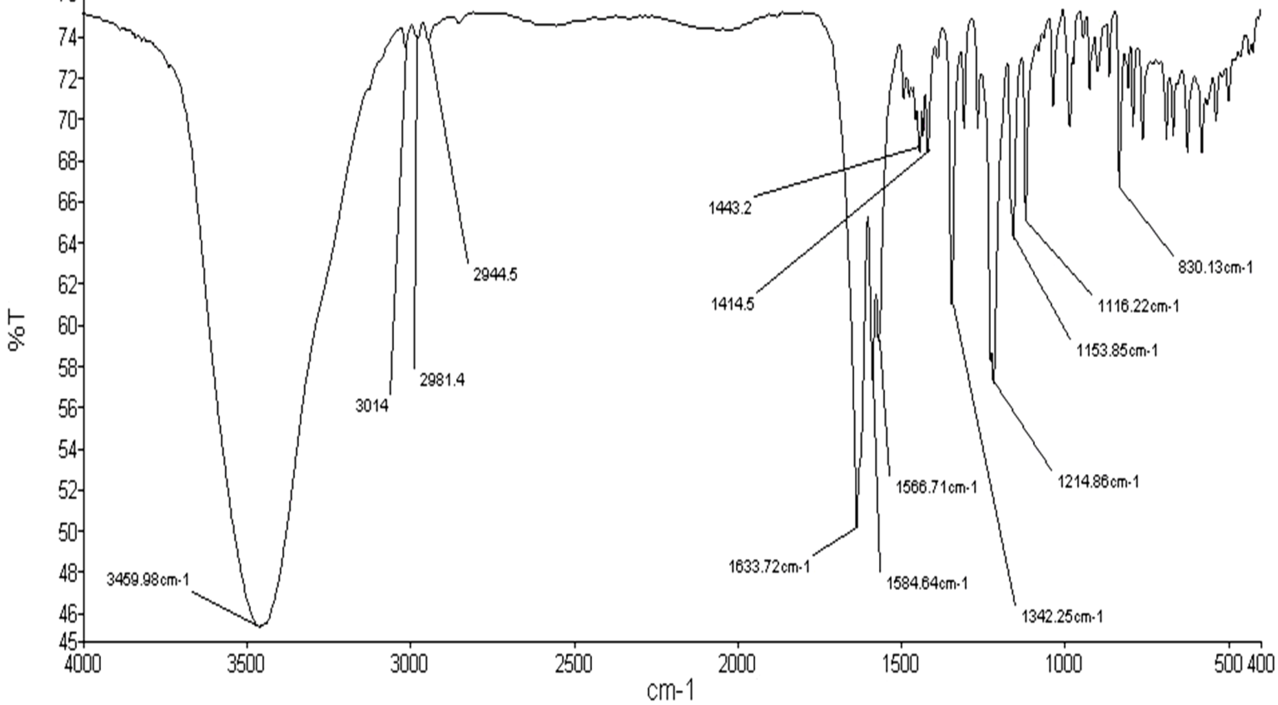

16. Fk2Cl (**16**)

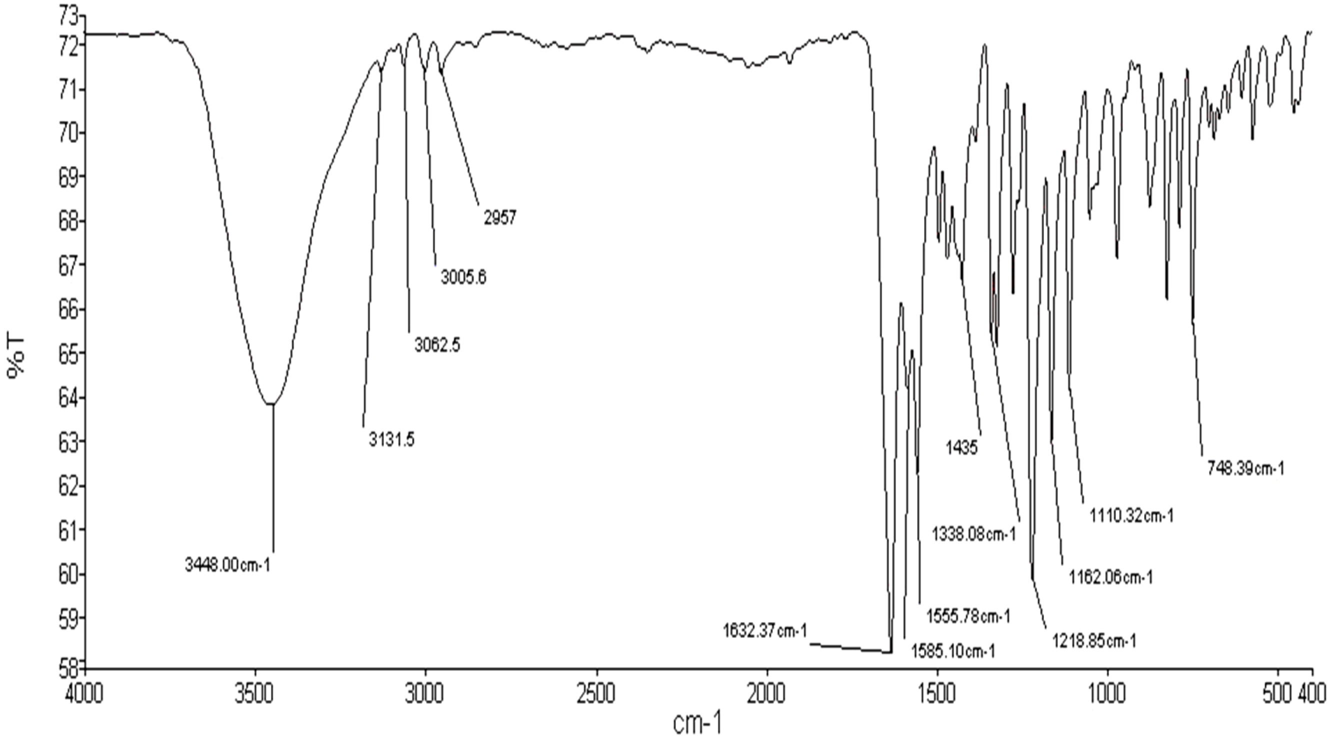

17. Fk4Cl (**17**)

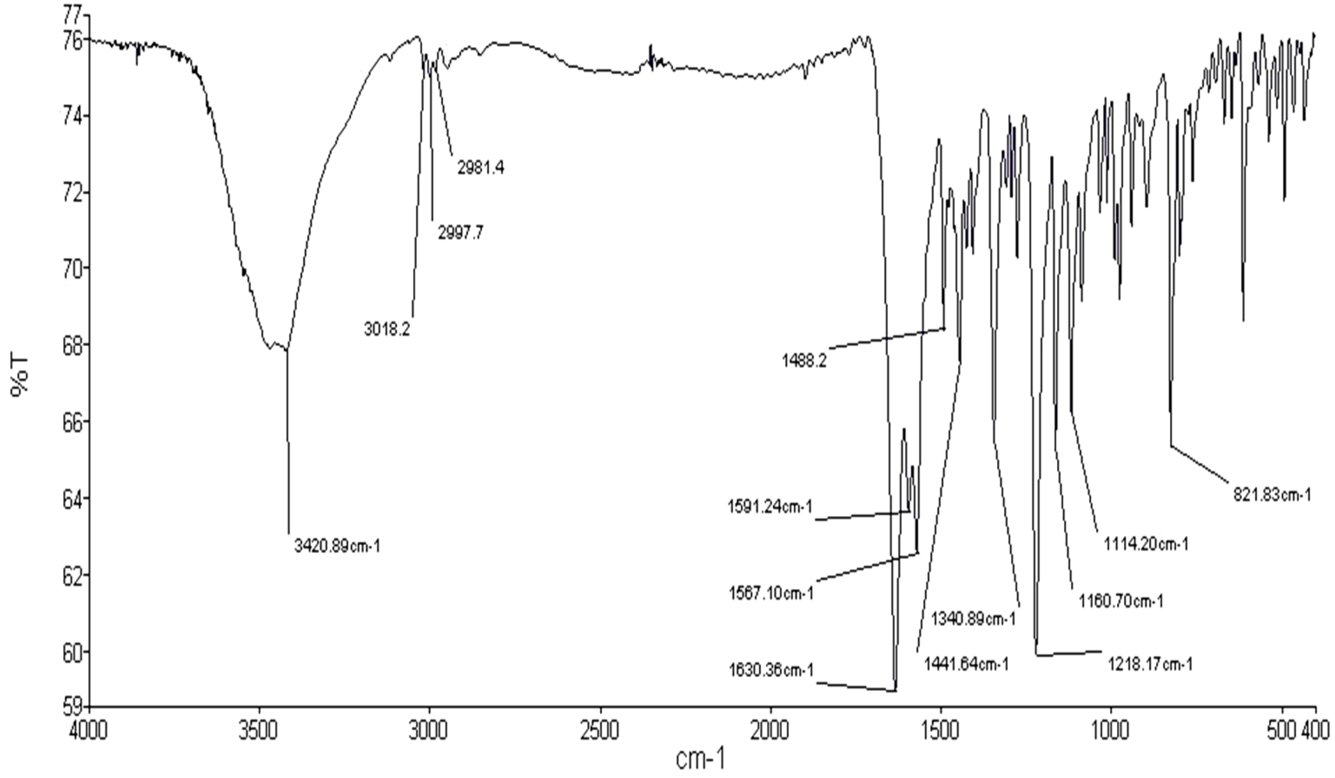

18. Fk4Br (**18**)

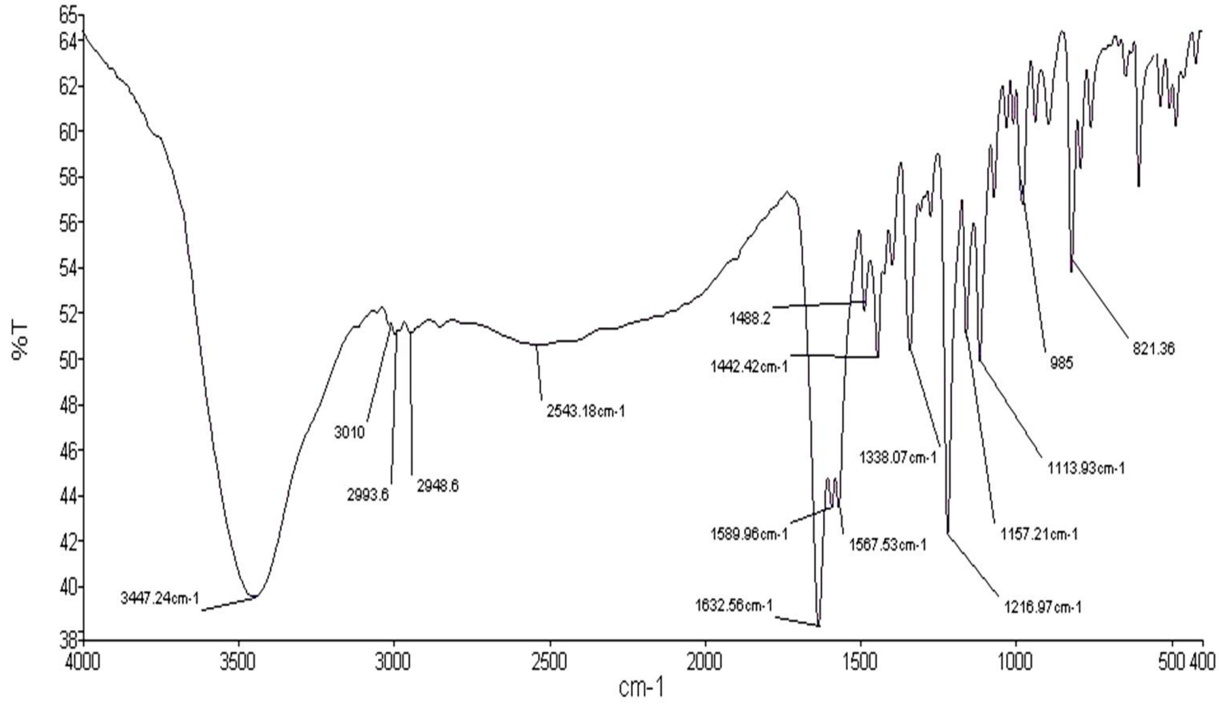

19. Fkav (**19**)

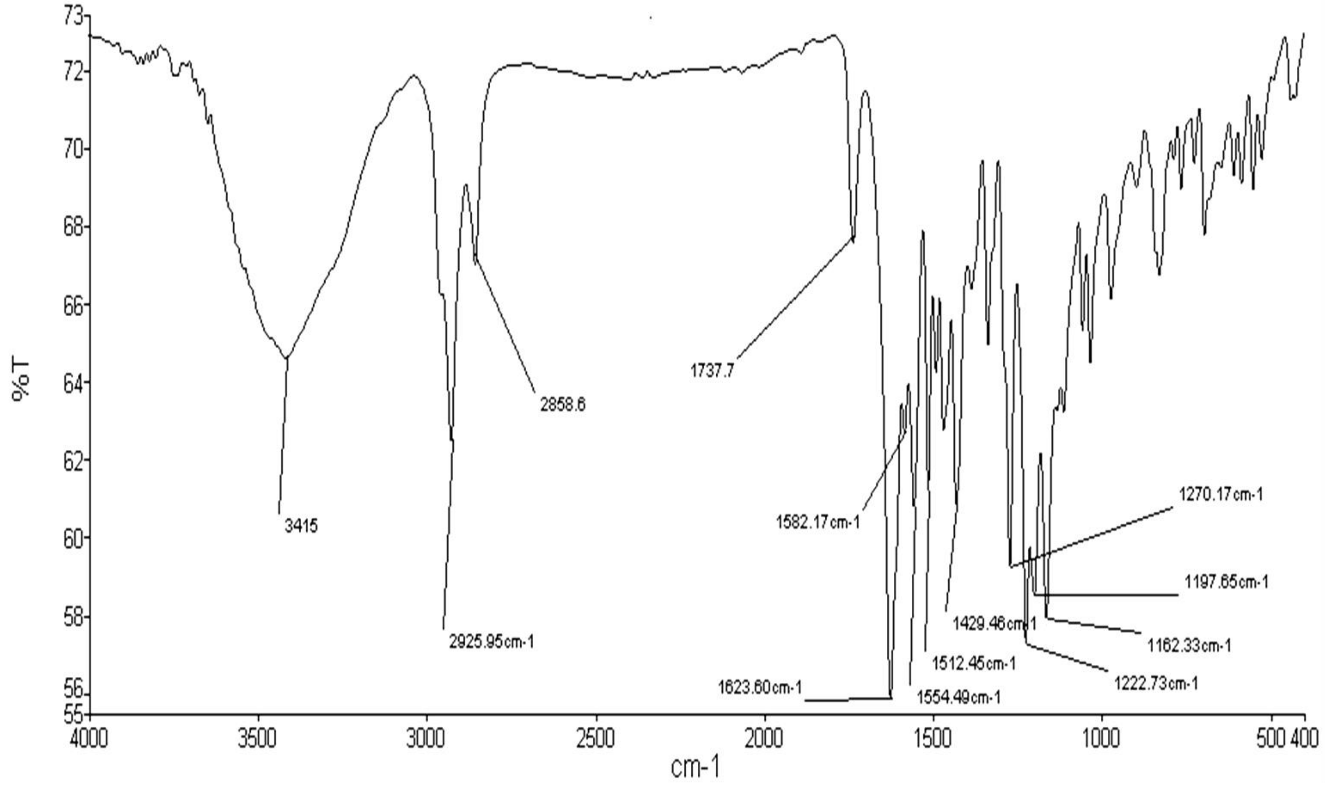

20. Fk3NO2 (**20**)

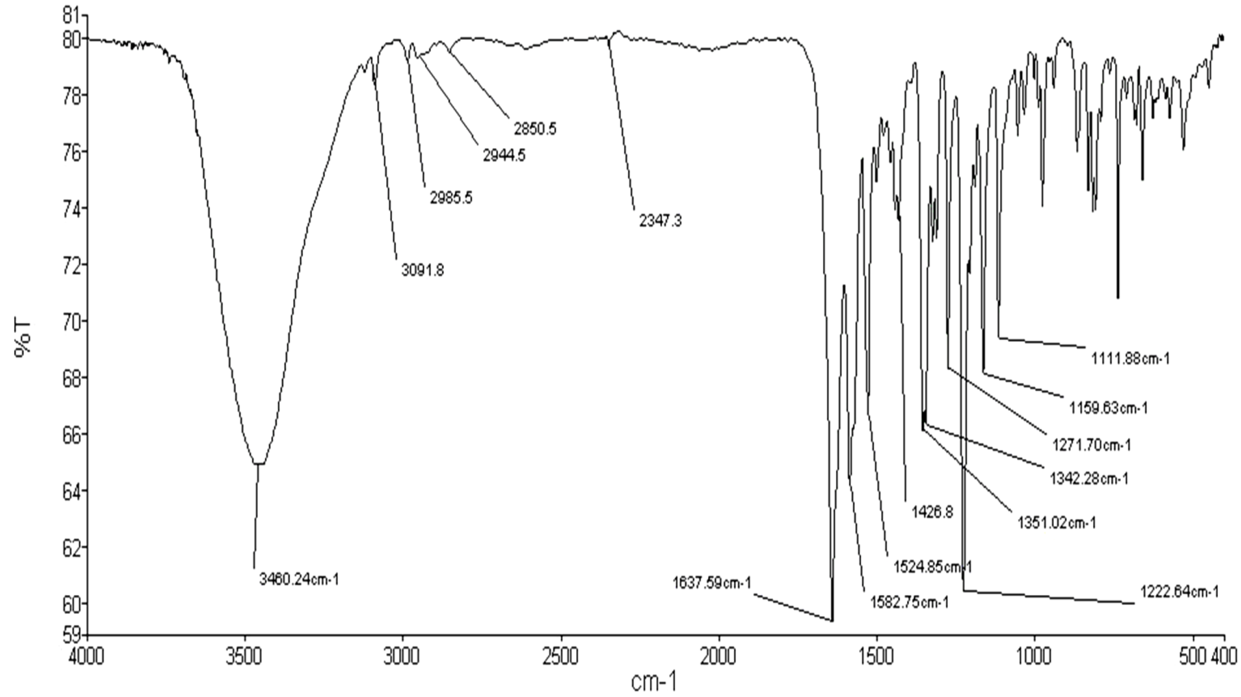

21. FkNM (**21**)

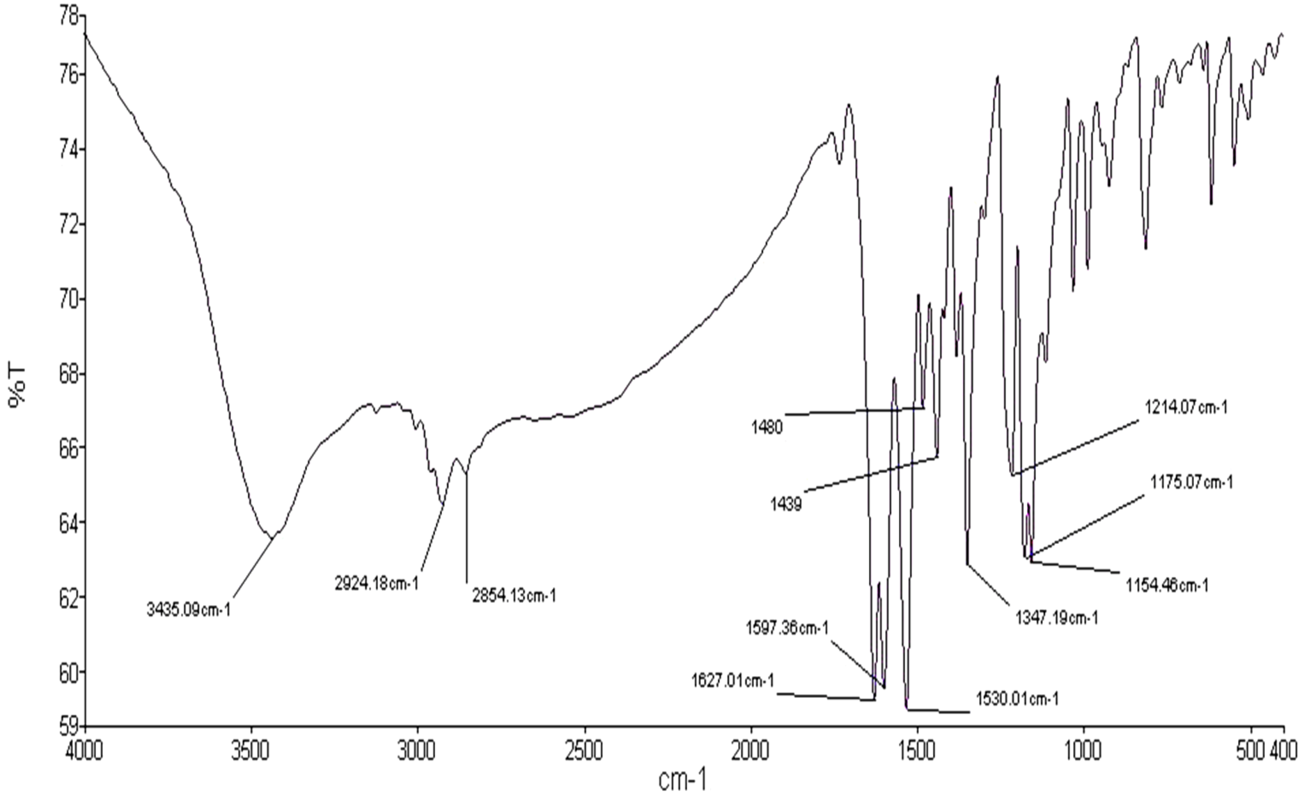

22. Fk5BS (**22**)

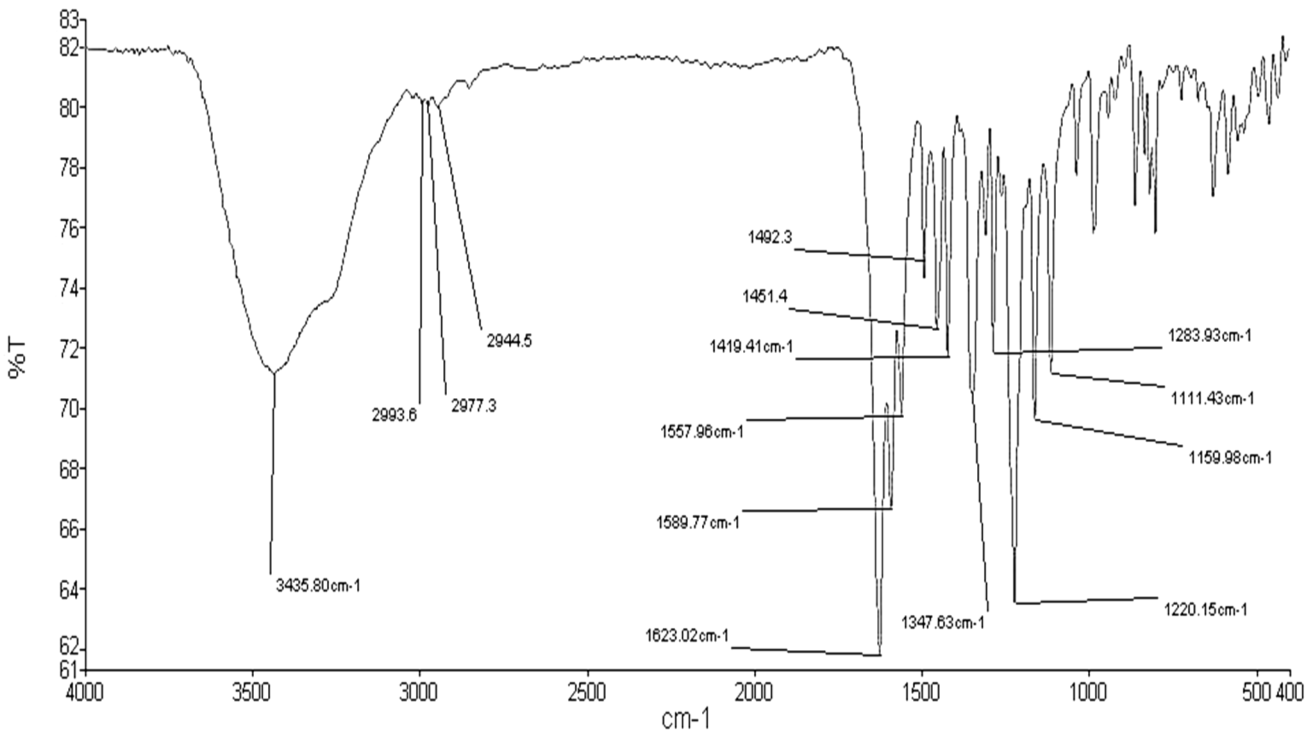

%T
cm-1
3435.80cm-1
2993.6
2977.3
2944.5
1492.3
1451.4
1419.41cm-1
1557.96cm-1
1589.77cm-1
1623.02cm-1
1347.63cm-1
1283.93cm-1
1111.43cm-1
1159.98cm-1
1220.15cm-1

23. Fk7ex (**23**)

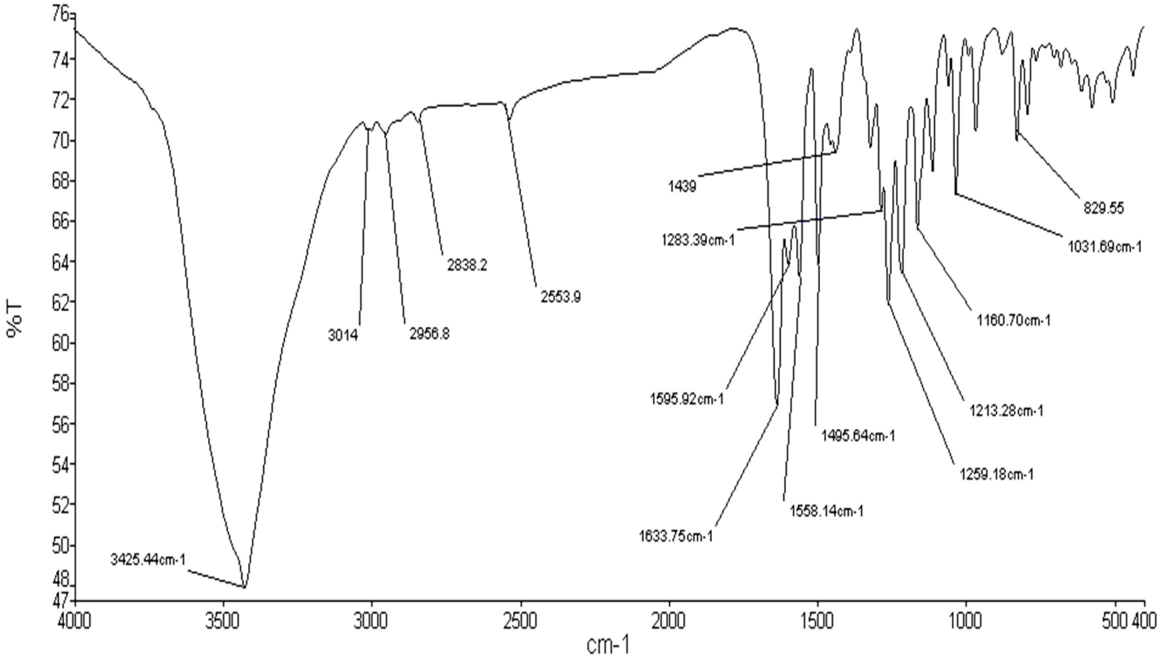

%T
cm-1
3425.44cm-1
3014
2956.8
2838.2
2553.9
1439
1283.39cm-1
1595.92cm-1
1633.75cm-1
1558.14cm-1
1495.64cm-1
1259.18cm-1
1213.28cm-1
1160.70cm-1
1031.69cm-1
829.55
