# Supplementary material for: Design, Synthesis and Docking Studies of Flavokawain B Type Chalcones and Their Cytotoxic Effects on MCF-7 and MDA-MB-231 Cell Lines
Source: Molecules. 2018 Mar 8;23(3):616. doi: 10.3390/molecules23030616 (PMC6017189; doi:10.3390/molecules23030616)

(*E*)-1-(2'-hydroxy-4',6'-dimethoxyphenyl)-3-phenylprop-2-en-1-one (**1**)

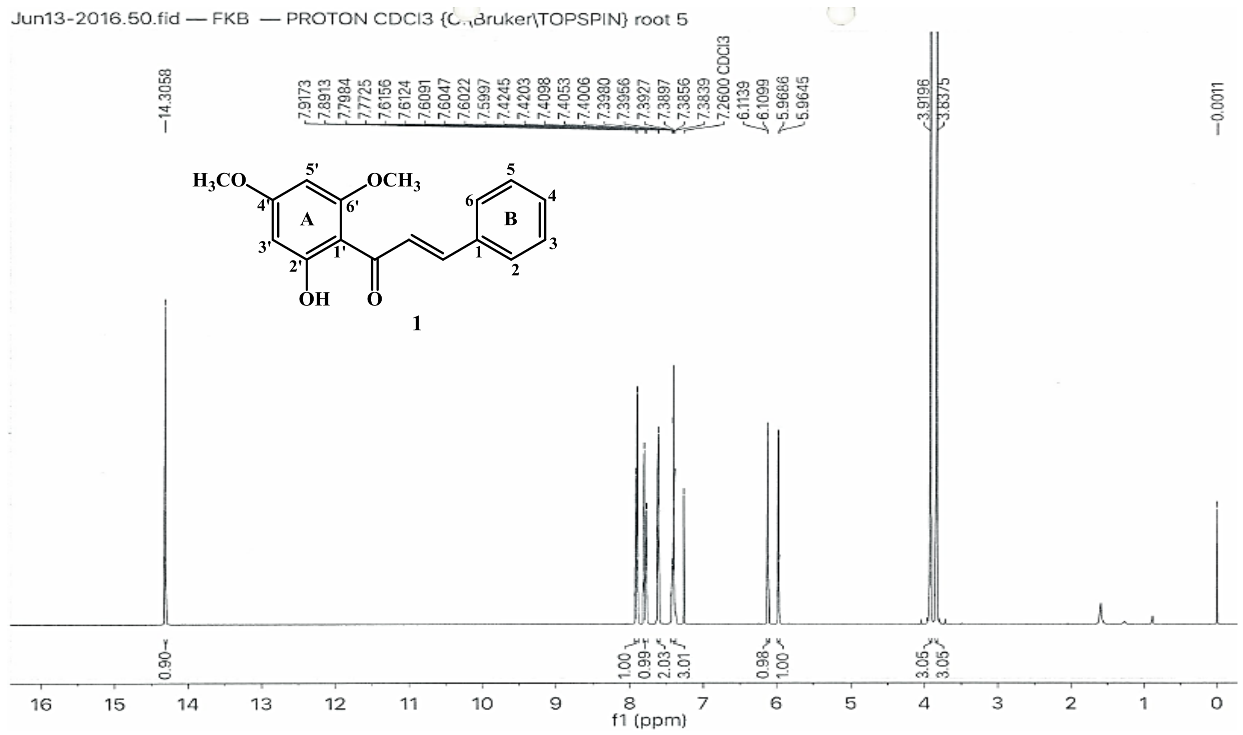

(*E*)-1-(2'-hydroxyl-4',6'-dimethoxyphenyl)-3-(4-methoxyphenyl)prop-2-en-1-one (**2**)

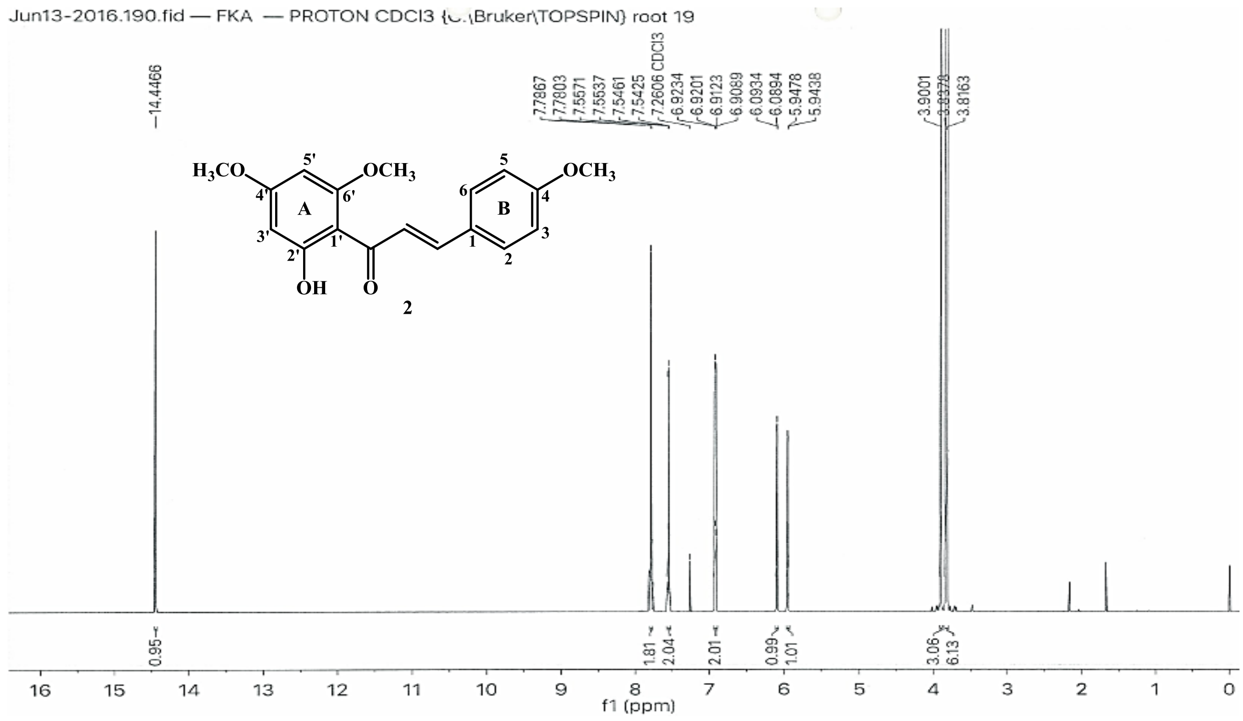

(*E*)-1-(2'-hydroxy-4',6'-dimethoxyphenyl)-3-(4-(methylthio)phenyl)prop-2-en-1-one (**3**)

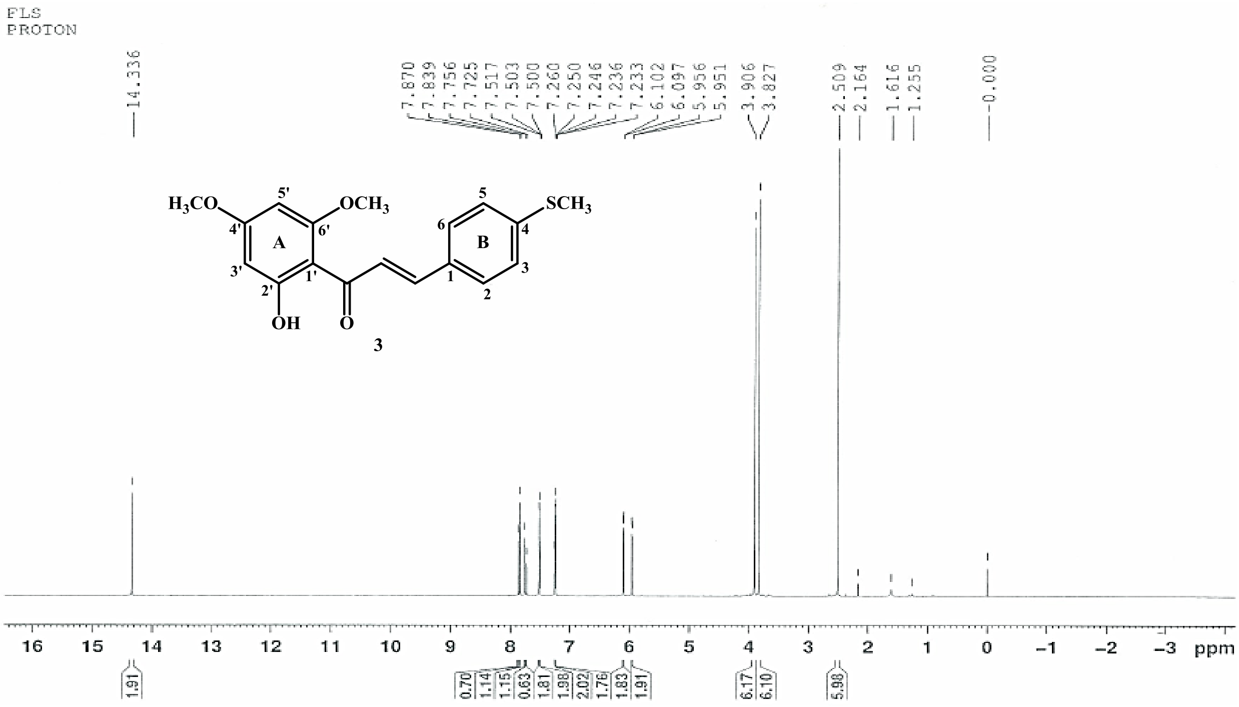

(*E*)-3-(2,3-dimethoxyphenyl)-1-(2'-hydroxy-4',6'-dimethoxyphenyl)prop-2-en-1-one (**4**)

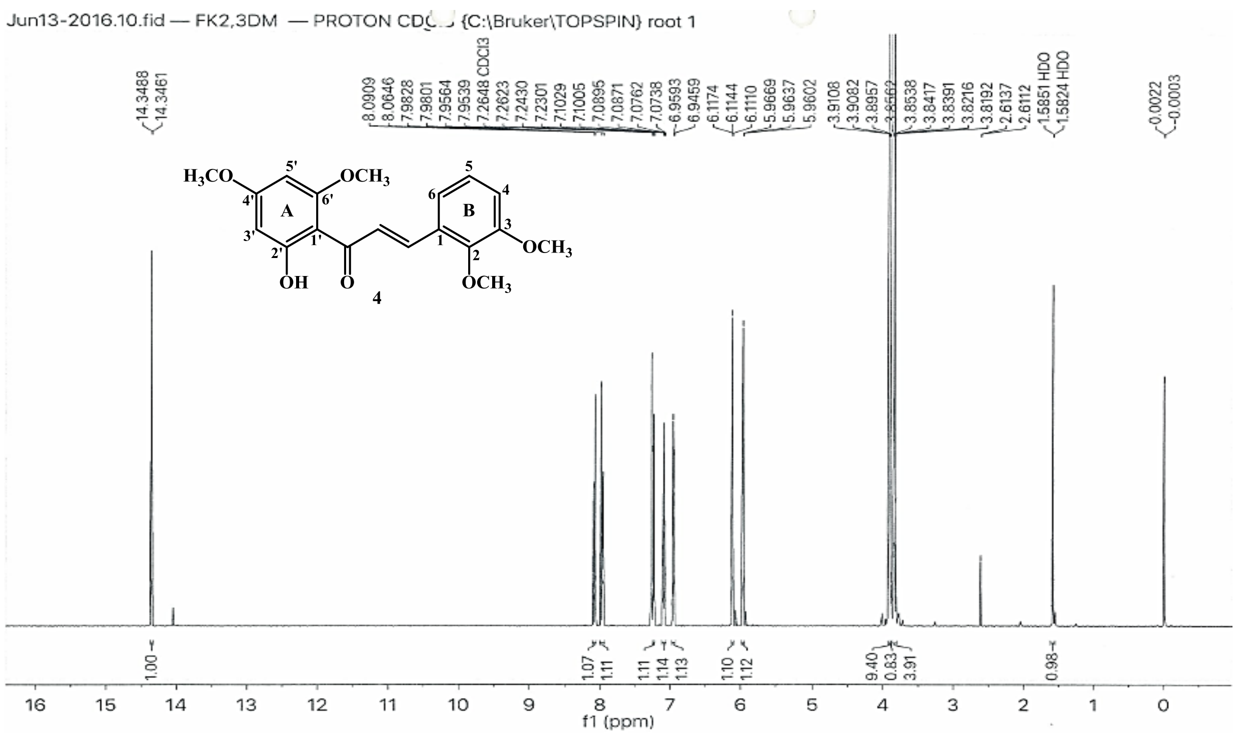

(*E*)-3-(2,4-dimethoxyphenyl)-1-(2'-hydroxy-4',6'-dimethoxyphenyl)prop-2-en-1-one (**5**)

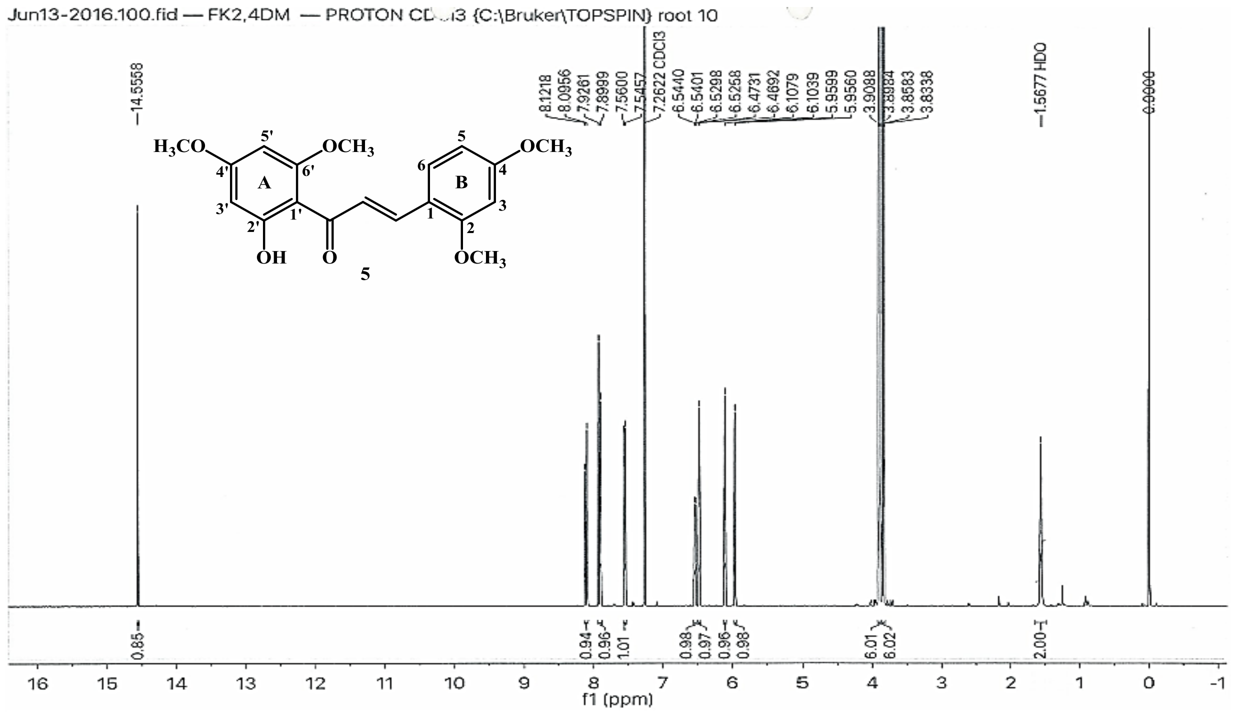

(*E*)-1-(2'-hydroxy-4',6'-dimethoxyphenyl)-3-(2,4,6-trimethoxyphenyl)prop-2-en-1-one (**6**)

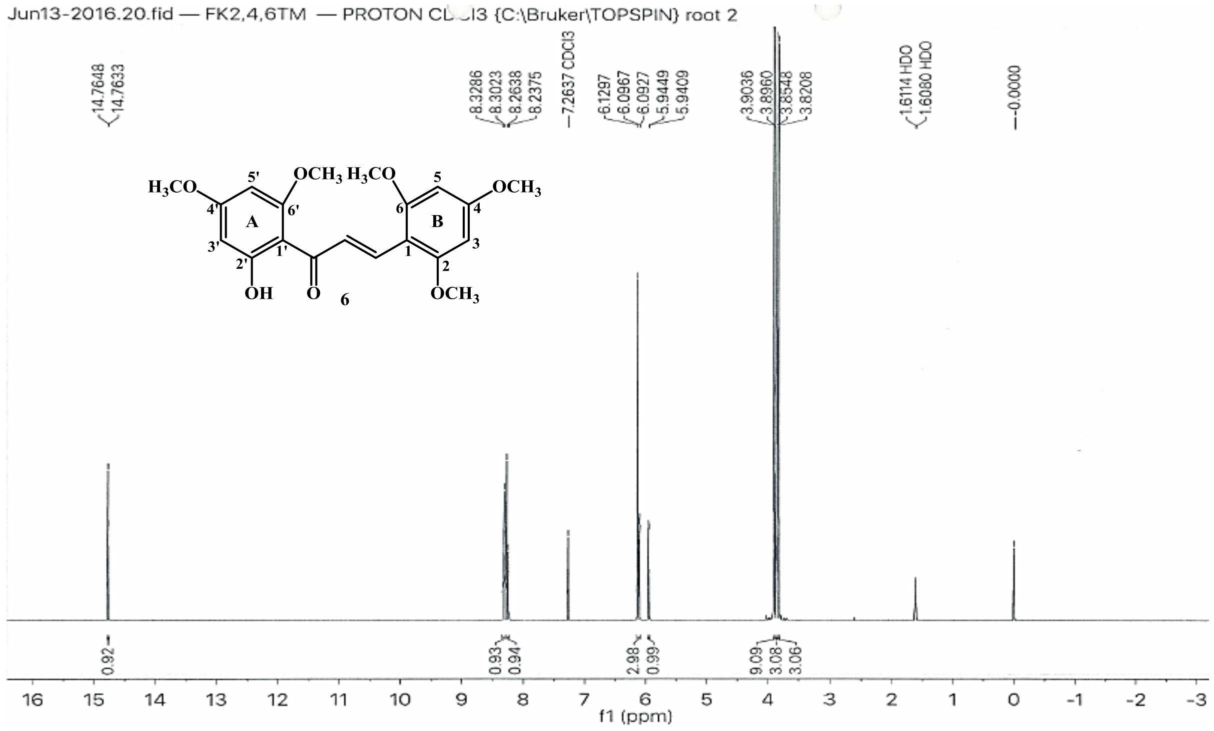

(*E*)-3-(3,4-dimethoxyphenyl)-1-(2'-hydroxy-4',6'-dimethoxyphenyl)prop-2-en-1-one (**7**)

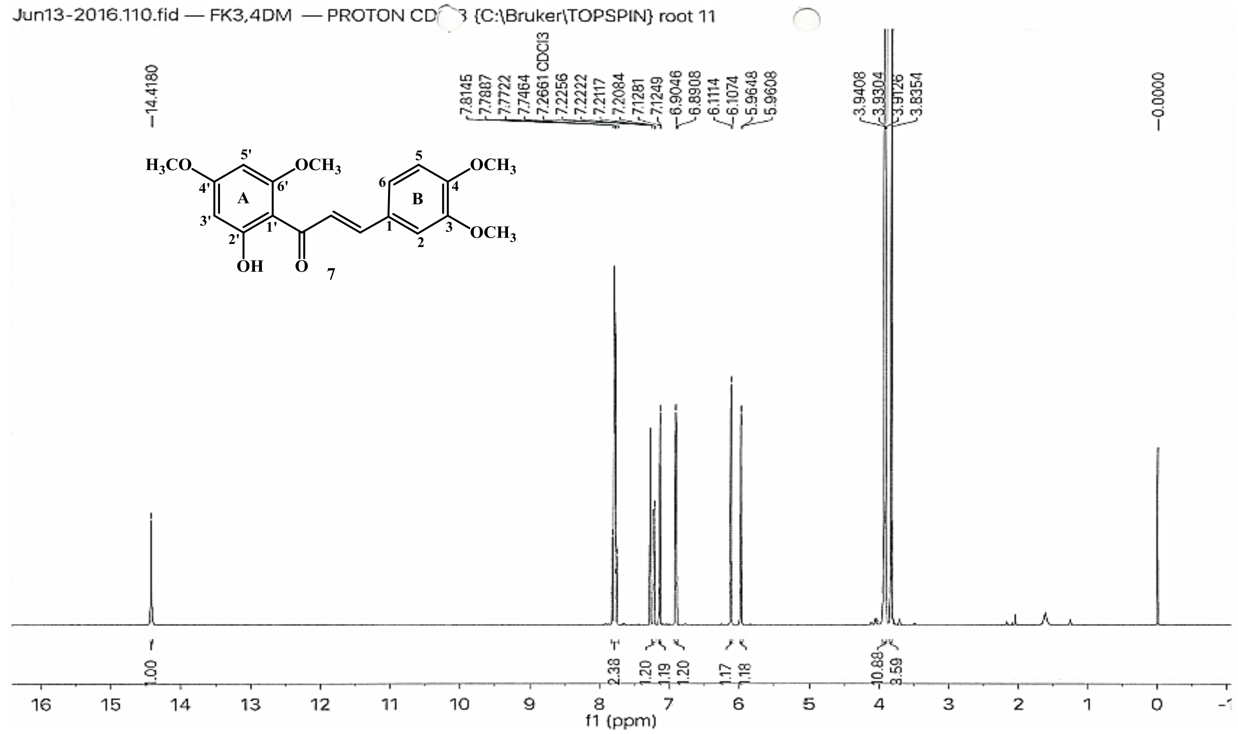

(*E*)-3-(2,5-dimethoxyphenyl)-1-(2'-hydroxy-4',6'-dimethoxyphenyl)prop-2-en-1-one (**8**)

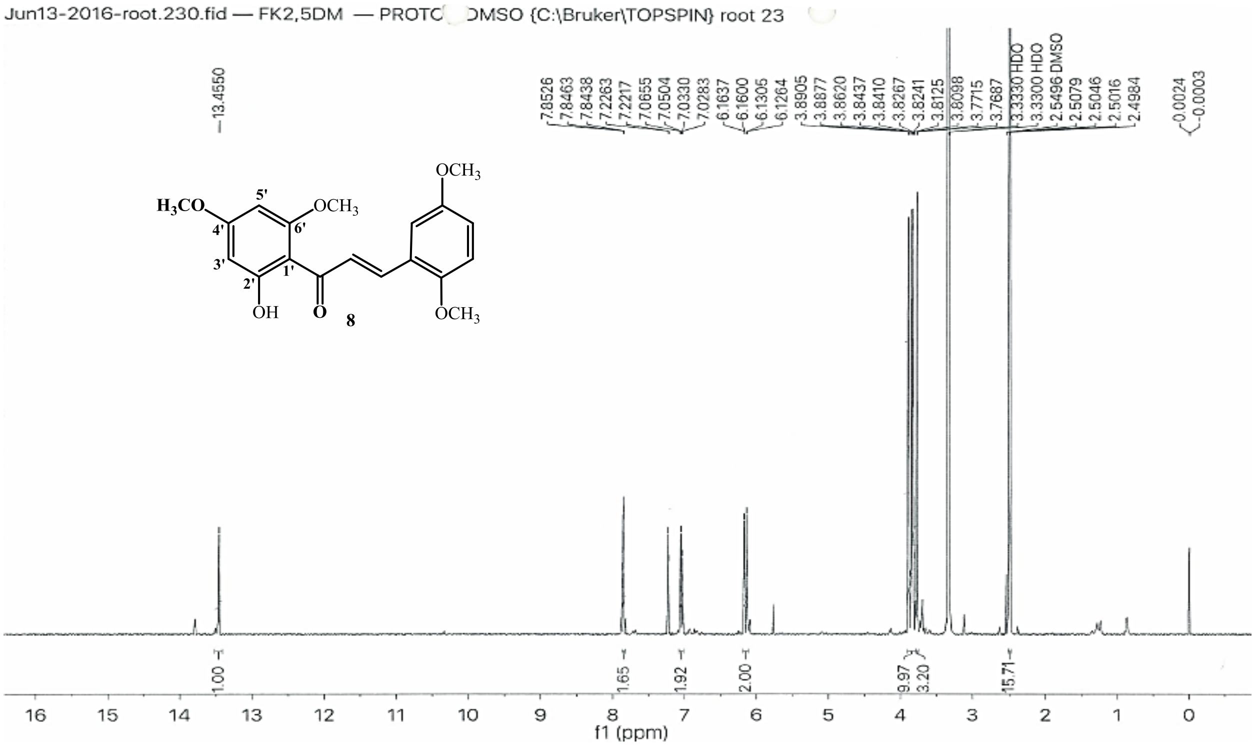

(*E*)-1-(2'-hydroxy-4',6'-dimethoxyphenyl)-3-(3-methoxyphenyl)prop-2-en-1-one (**9**)

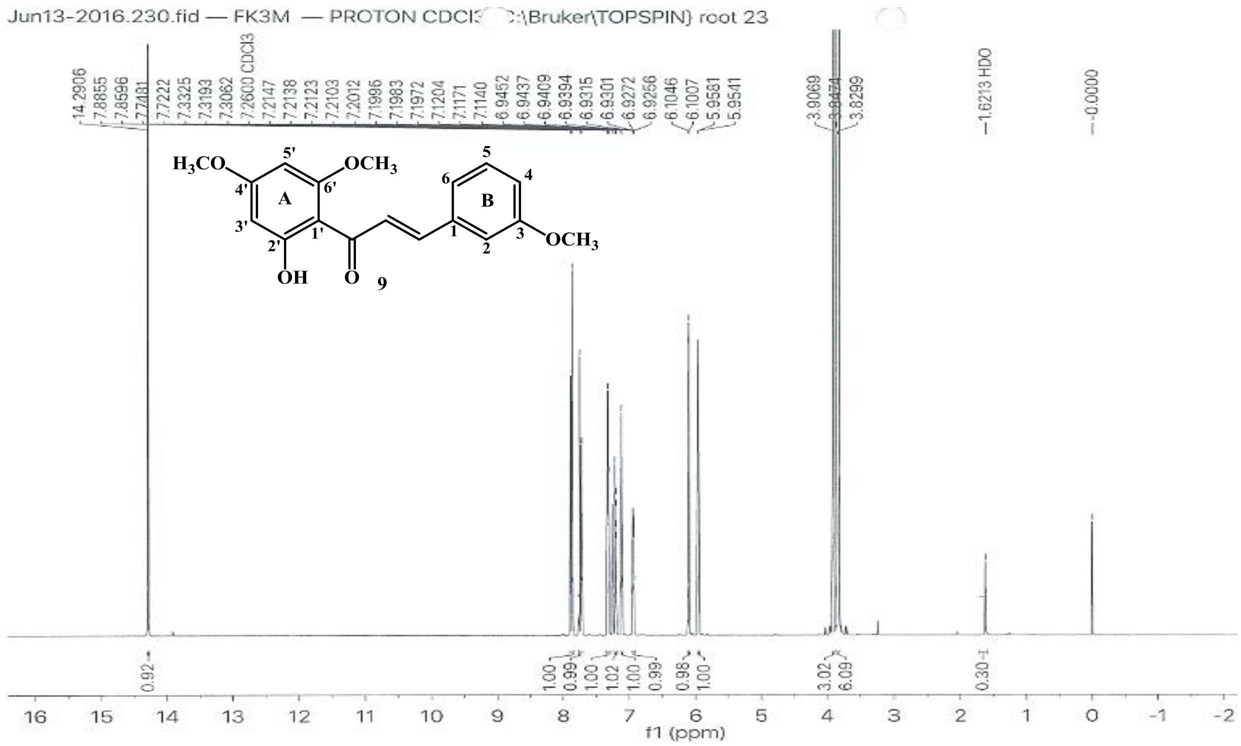

(*E*)-3-(3,5-dimethoxyphenyl)-1-(2'-hydroxy-4',6'-dimethoxyphenyl)prop-2-en-1-one (**10**)

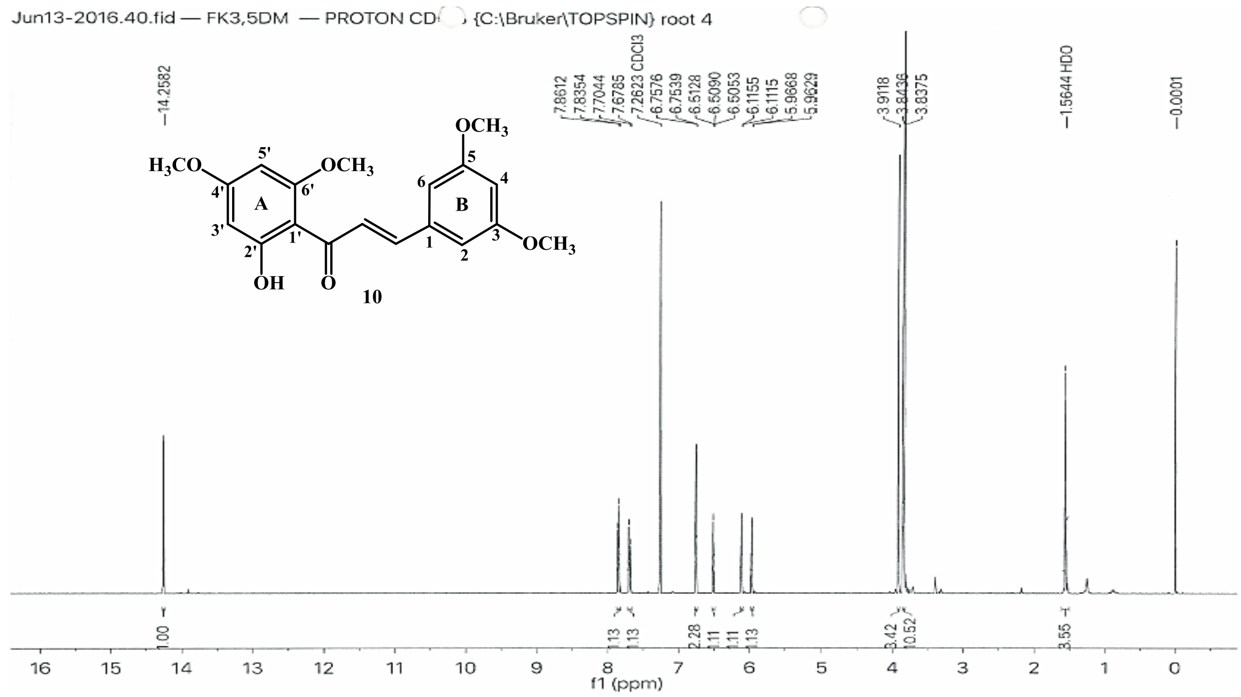

(*E*)-1-(2'-hydroxy-4',6'-dimethoxyphenyl)-3-(p-tolyl)prop-2-en-1-one (**11**)

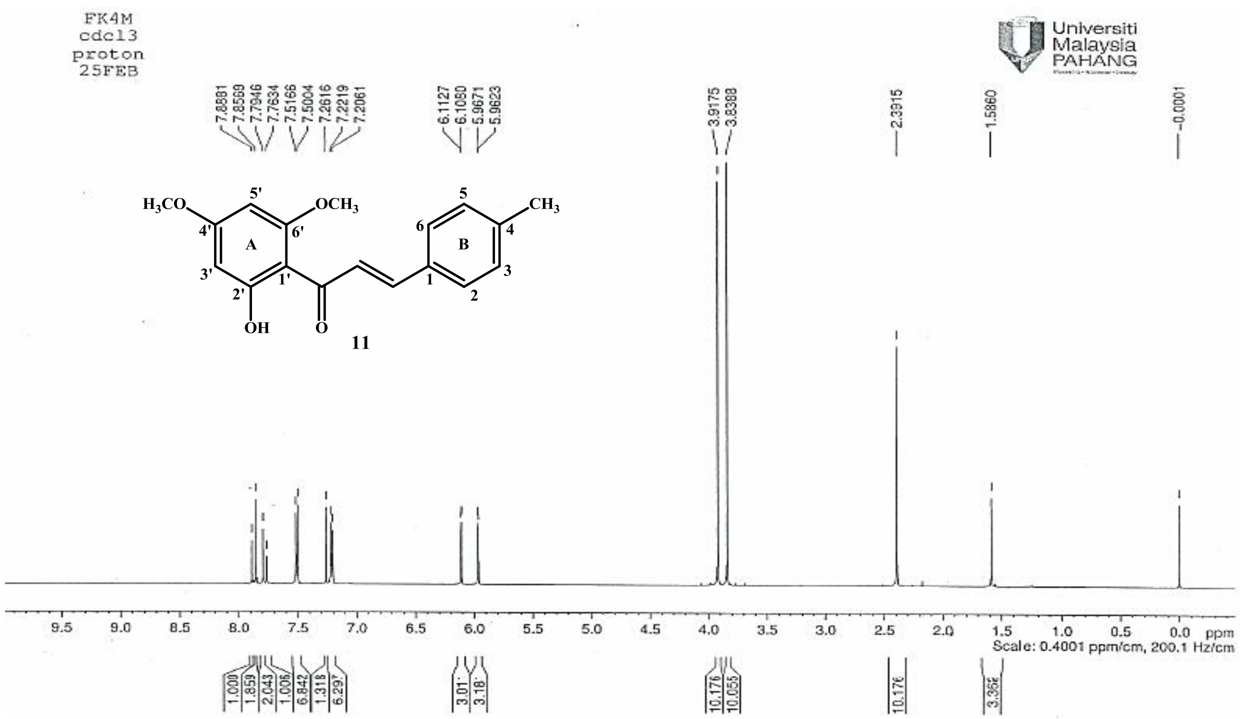

(*E*)-1-(2'-hydroxy-4',6'-dimethoxyphenyl)-3-(2-methoxyphenyl)prop-2-en-1-one (**12**)

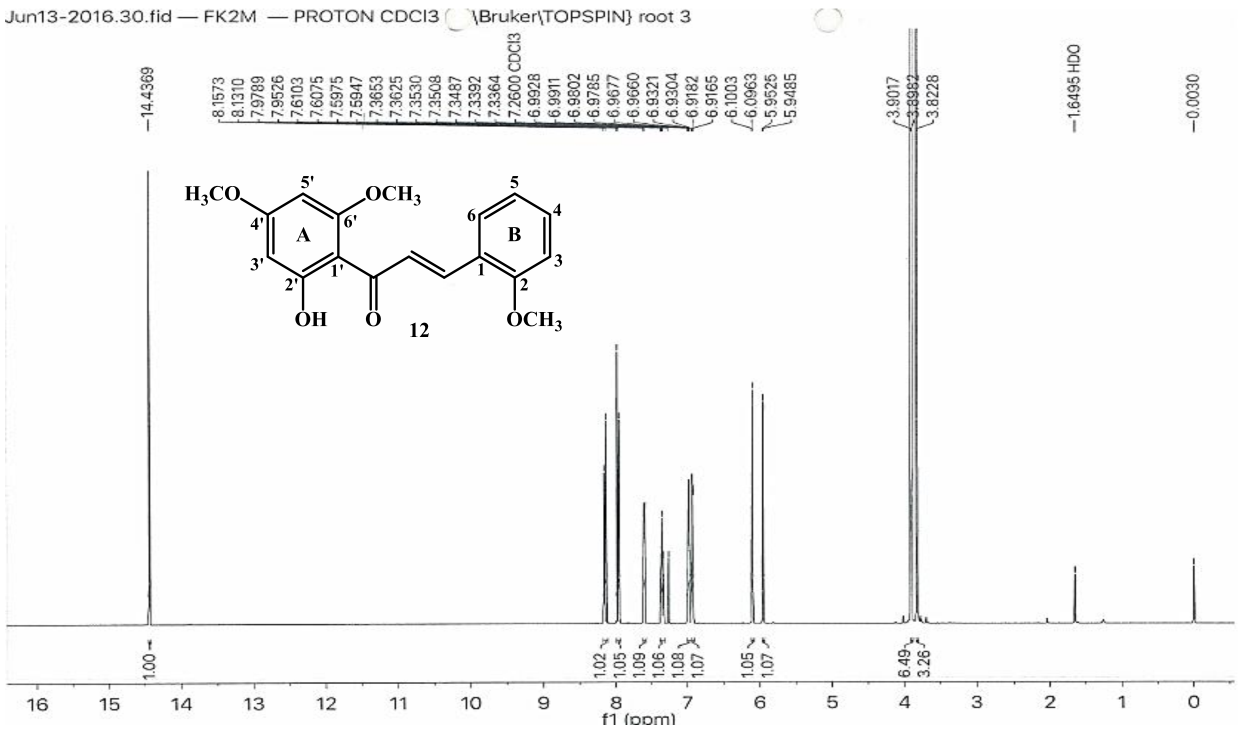

(*E*)-3-(2-fluorophenyl)-1-(2'-hydroxy-4',6'-dimethoxyphenyl)prop-2-en-1-one (**13**)

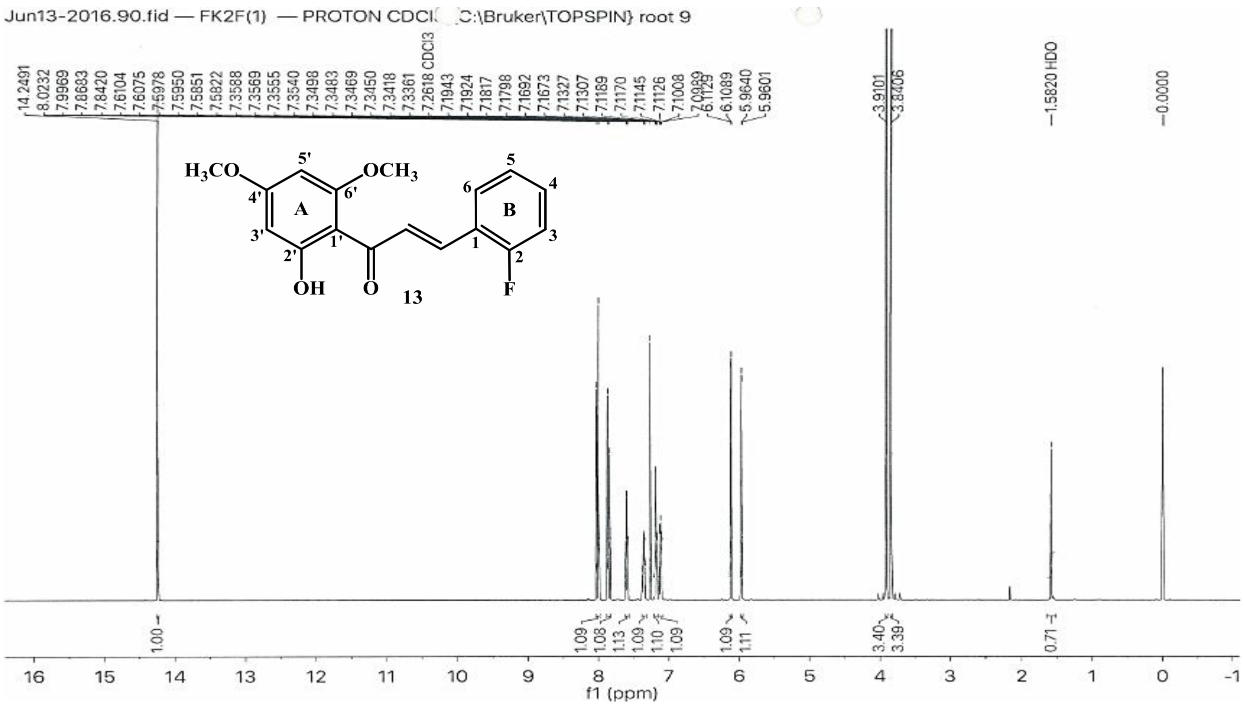

(*E*)-3-(4-fluorophenyl)-1-(2'-hydroxy-4',6'-dimethoxyphenyl)prop-2-en-1-one (**14**)

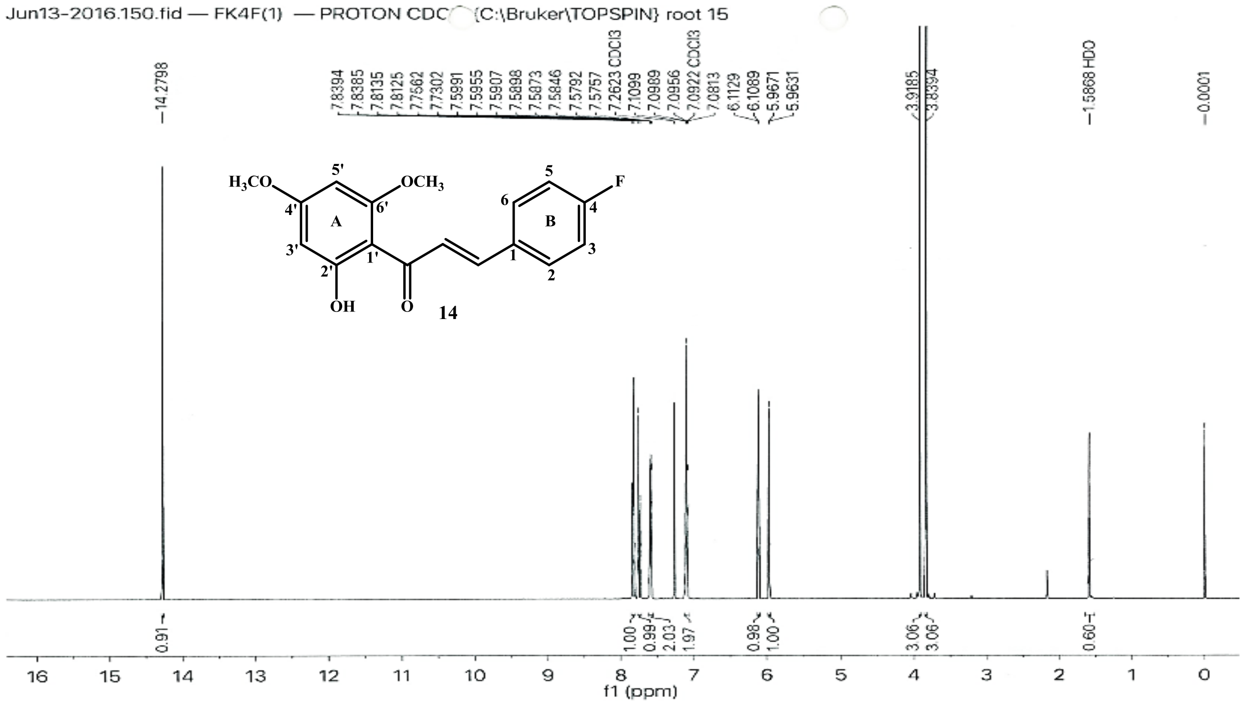

(*E*)-3-(3-chlorophenyl)-1-(2'-hydroxy-4',6'-dimethoxyphenyl)prop-2-en-1-one (**15**)

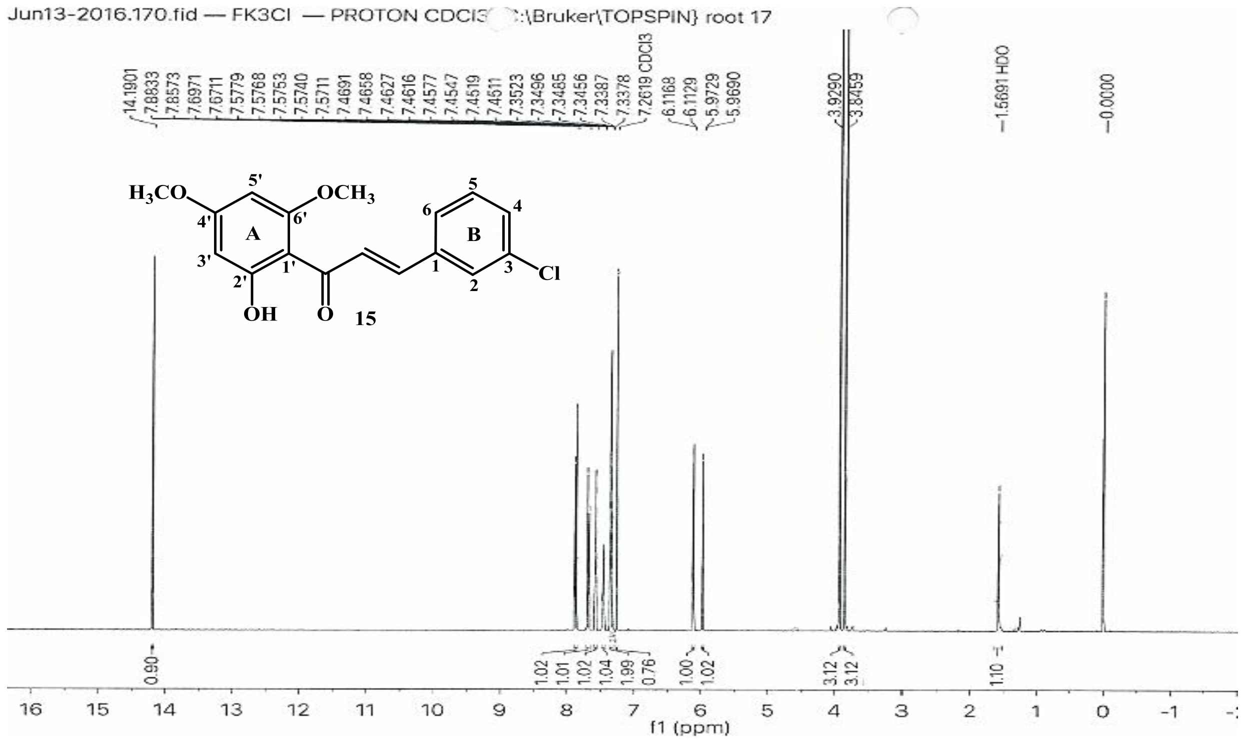

(*E*)-3-(2-chlorophenyl)-1-(2'-hydroxy-4',6'-dimethoxyphenyl)prop-2-en-1-one (**16**)

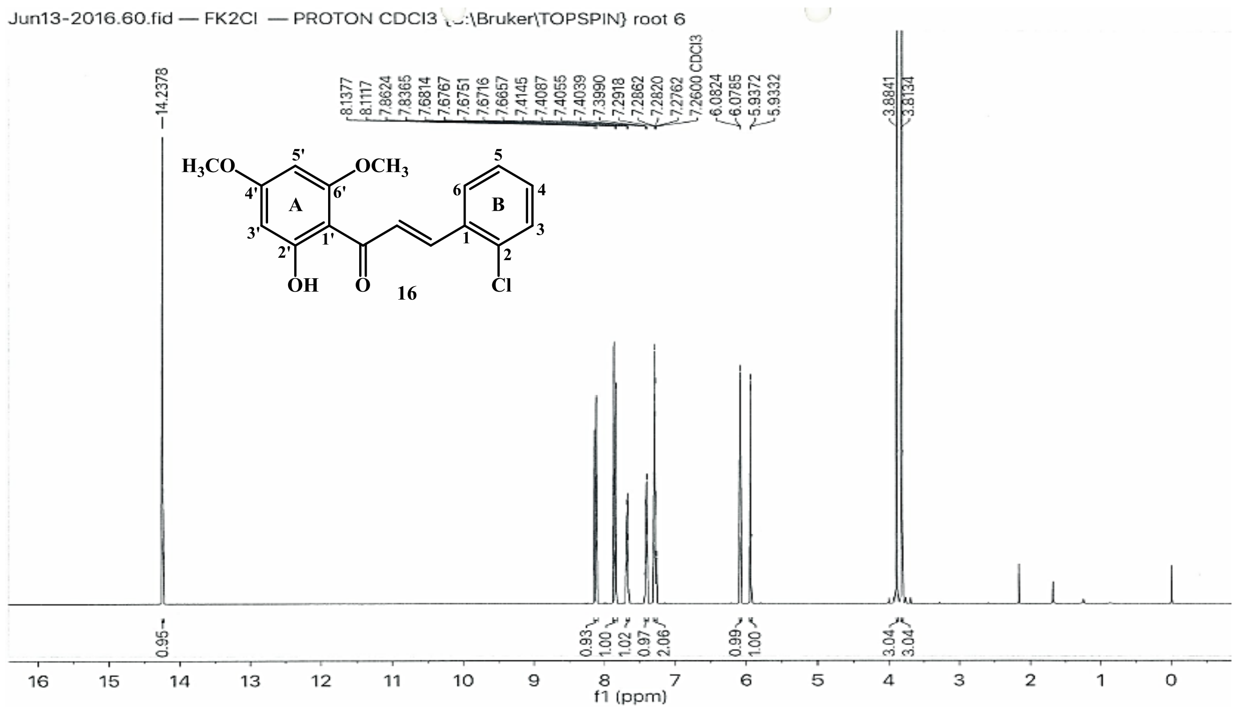

(*E*)-3-(4-chlorophenyl)-1-(2'-hydroxy-4',6'-dimethoxyphenyl)prop-2-en-1-one (**17**)

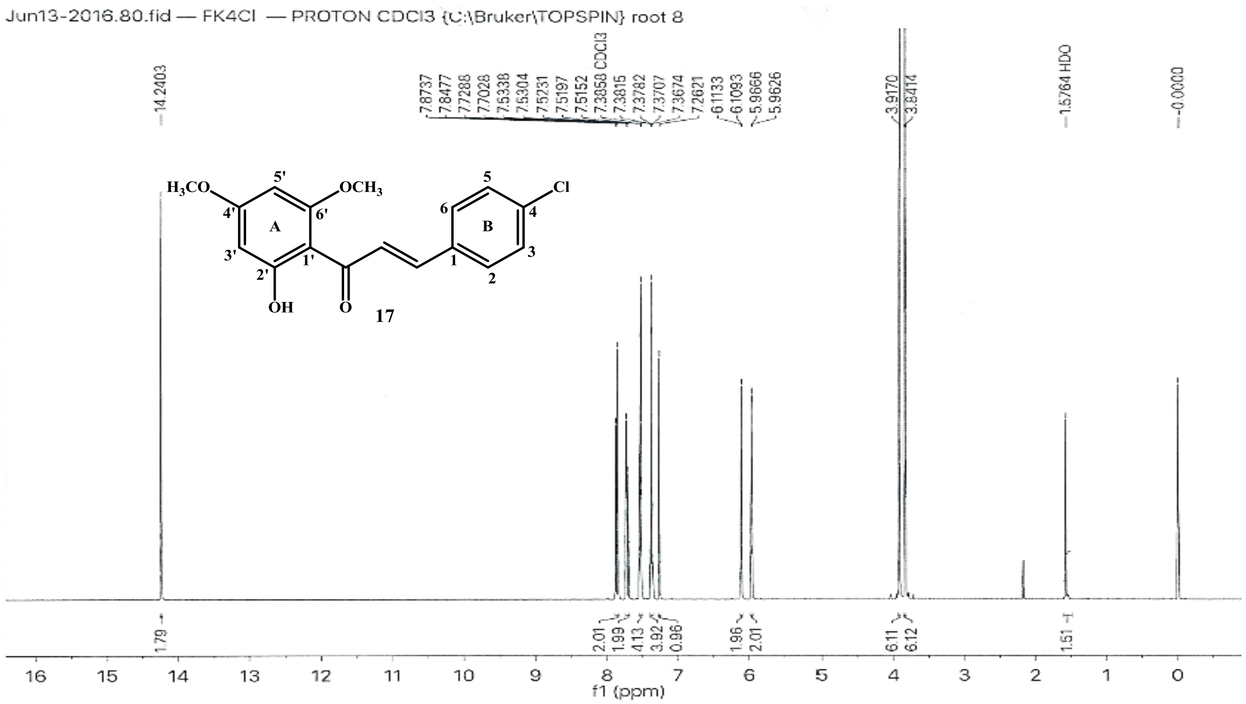

(*E*)-3-(4-bromophenyl)-1-(2'-hydroxy-4',6'-dimethoxyphenyl)prop-2-en-1-one (**18**)

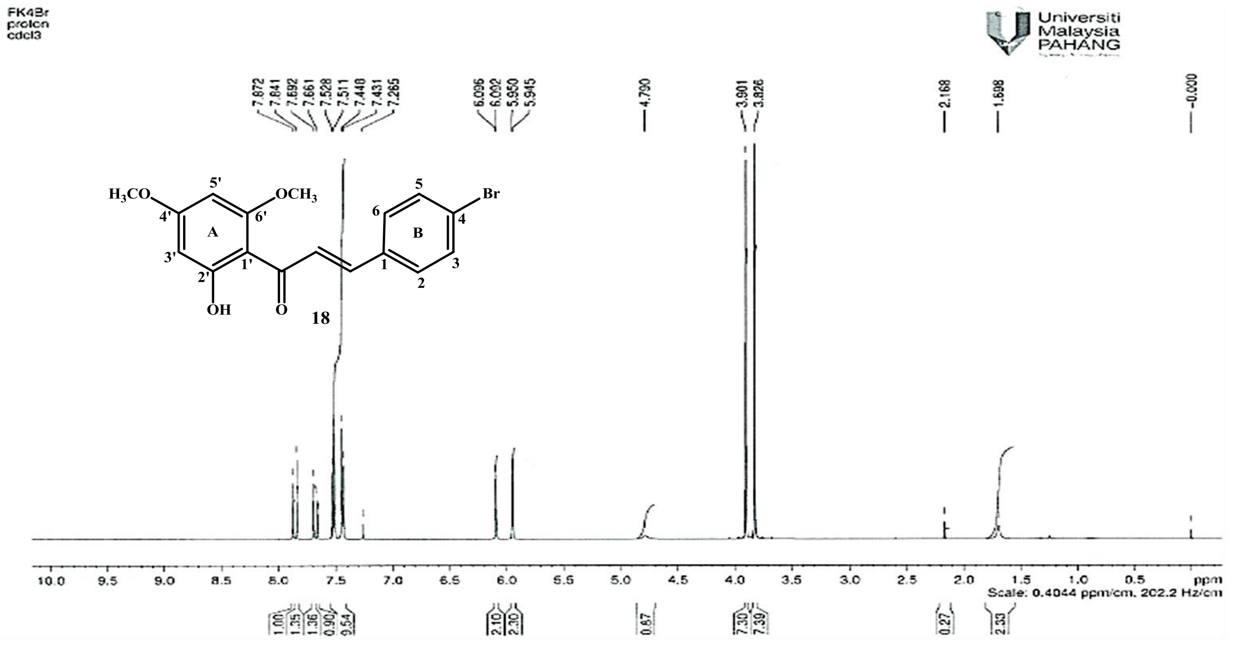

(*E*)-3-(4-hydroxy-3-methoxyphenyl)-1-(2'-hydroxy-4',6'-dimethoxyphenyl)prop-2-en-1-one (**19**)

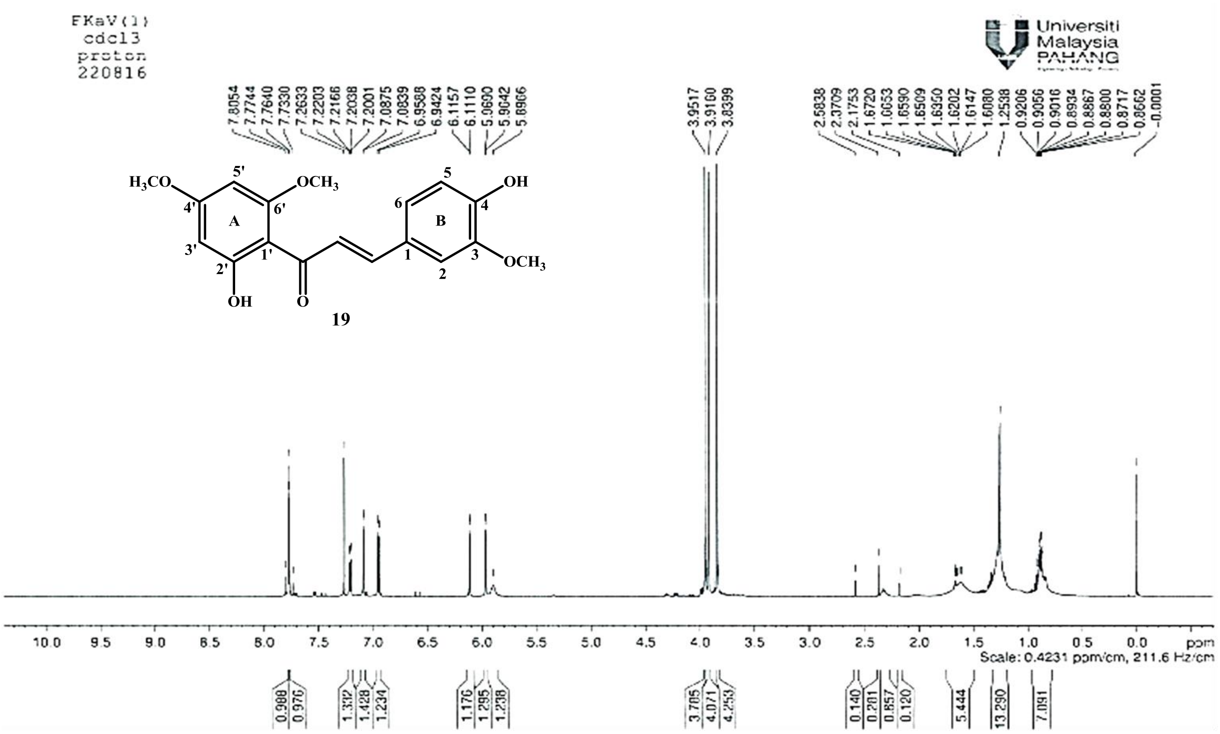

(*E*)-1-(2'-hydroxy-4',6'-dimethoxyphenyl)-3-(3-nitrophenyl)prop-2-en-1-one (**20**)

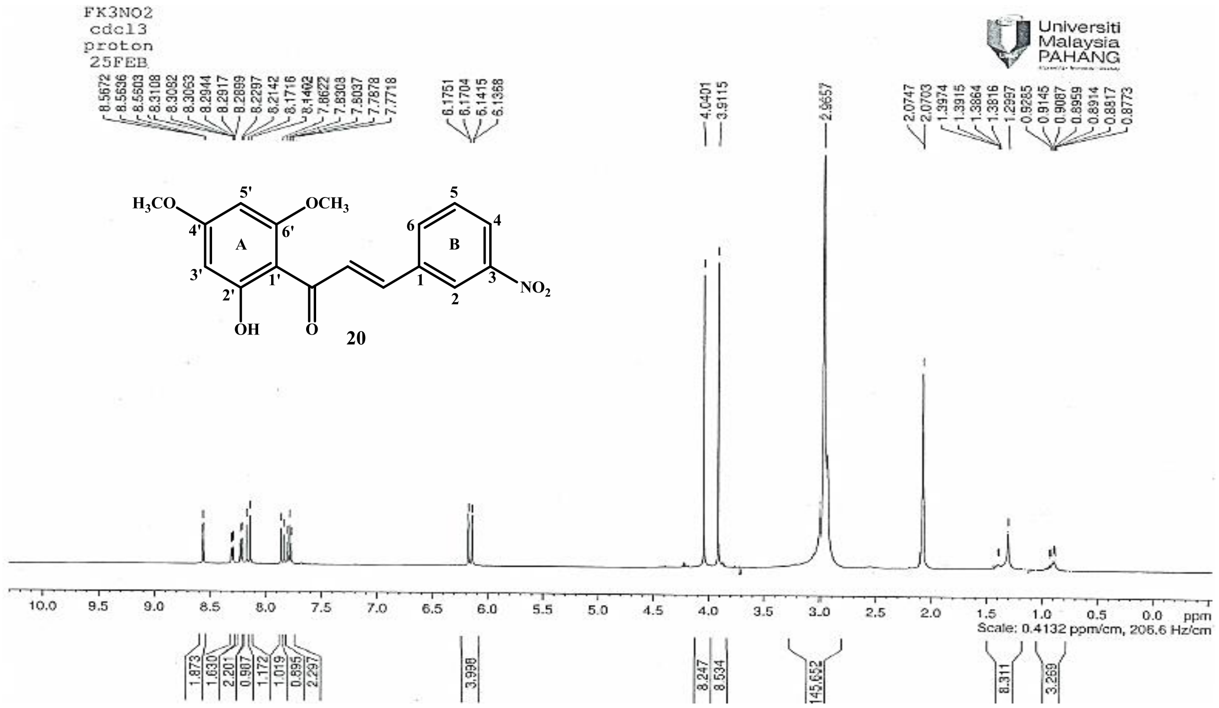

(*E*)-3-(4-(dimethylamino)phenyl)-1-(2'-hydroxy-4',6'-dimethoxyphenyl)prop-2-en-1-one (**21**)

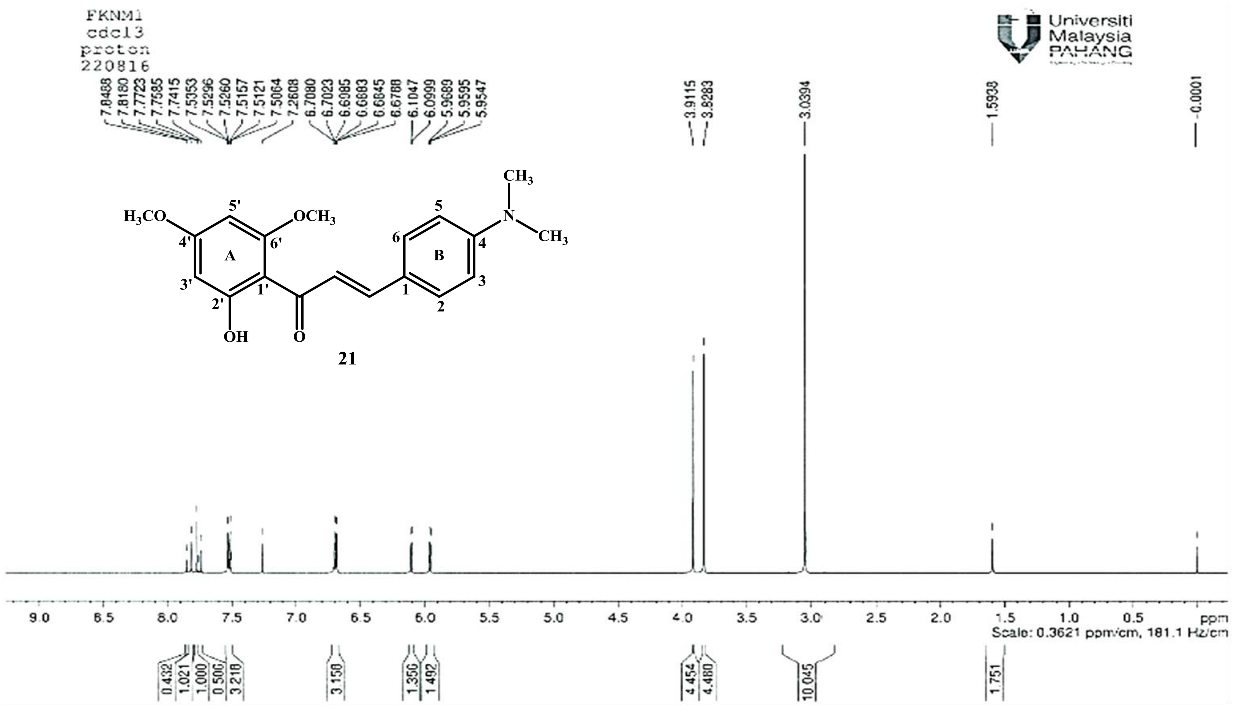

(*E*)-3-(5-bromo-2-hydroxyphenyl)-1-(2'-hydroxy-4',6'-dimethoxyphenyl)prop-2-en-1-one (**22**)

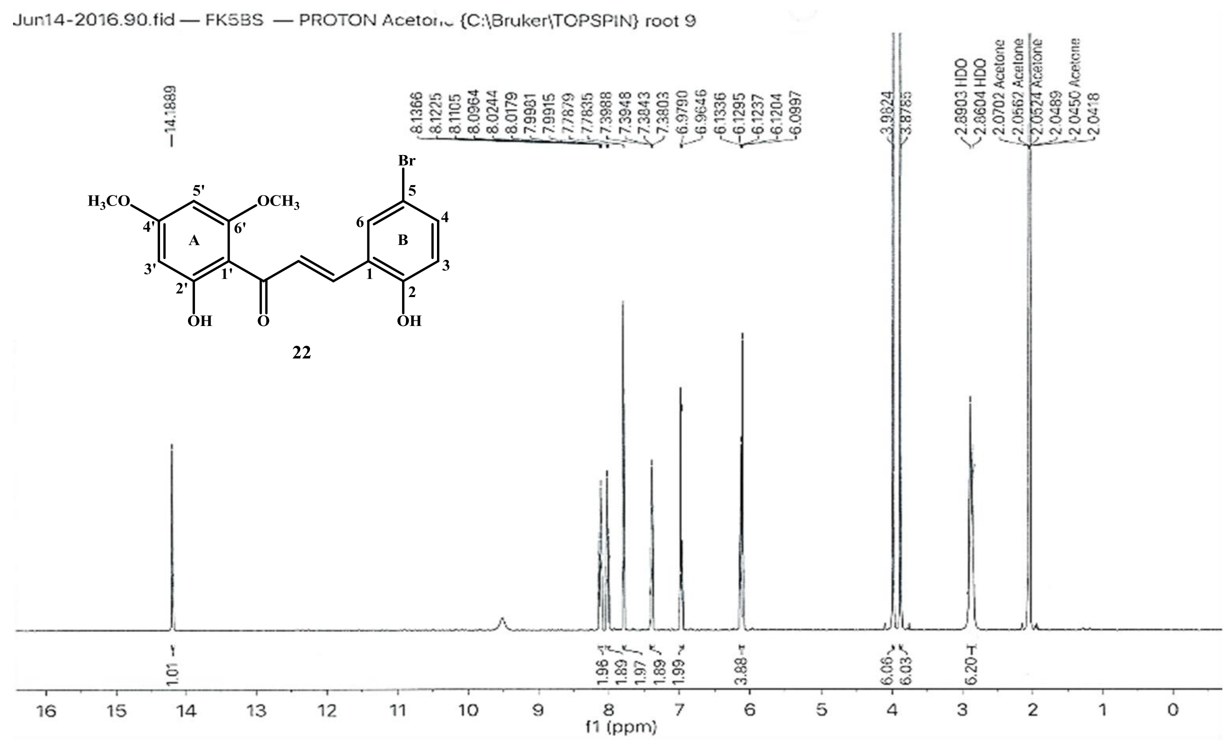

(*E*)-3-(2-bromo-3-hydroxy-4-methoxyphenyl)-1-(2'-hydroxy-4',6'-dimethoxyphenyl)prop-2-en-1-one (**23**)

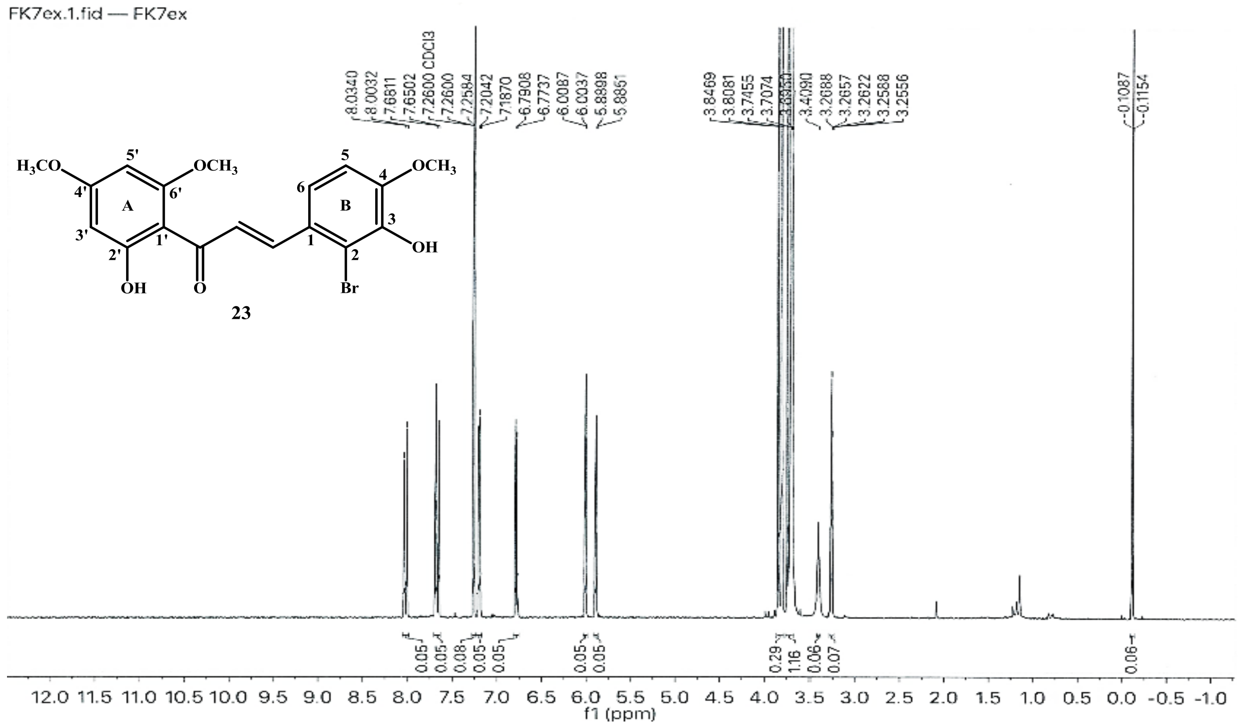

Supplement: Supplementary file 1 [file molecules-23-00616-s001.zip › NMR SPECTRA (supplementary data).pdf]
