# Supplementary material for: Design, Synthesis and Docking Studies of Flavokawain B Type Chalcones and Their Cytotoxic Effects on MCF-7 and MDA-MB-231 Cell Lines
Source: Molecules. 2018 Mar 8;23(3):616. doi: 10.3390/molecules23030616 (PMC6017189; doi:10.3390/molecules23030616)
Supplement: Supplementary file 1 [file molecules-23-00616-s001.zip › Supplementary data-docking 9-3-2018.pdf]

molecules

Supplementary data

Design, Synthesis and Docking Studies of Flavokawain B Type Chalcones and Their Cytotoxic Effects on MCF-7 and MDA-MB-231 Cell Lines

Addila Abu Bakar [1], Muhammad Nadeem Akhtar [1,*], Norlaily Mohd Ali [2], Swee Keong Yeap [3], Ching Kheng Quah [4], Wan-Sin Loh [4], Noorjahan Banu Alitheen [5,*], Seema Zareen [1], Zaheer Ul-Haq [6] and Syed Adnan Ali Shah [7]

[1] Faculty of Industrial Sciences & Technology, Universiti Malaysia Pahang, Lebuhraya Tun Razak 26300, Kuantan Pahang, Malaysia; addilaabubakar@gmail.com (A.A.B.); seema@ump.edu.my (S.Z.)
[2] Faculty of Medicine and Health Sciences, Univesiti Tunku Abdul Rahman, Sungai Long, 43400, Selangor, Malaysia; norlailyma@gmail.com
[3] Chine-ASEAN College of Marine Sciences, Xiamen University Malaysia, Jalan Sunsuria, Bandar Sunsuria, Sepang 43900, Selangor, Malaysia; skyeap2005@gmail.com
[4] X-ray Crystallography Unit, School of Physics, Universiti Sains Malaysia, Penang 11800 USM, Malaysia; ck.quah@hotmail.com (C.K.Q.); wansin_loh@live.com (W.-S.L.)
[5] Department of Cell and Molecular Biology, Faculty of Biotechnology and Biomolecular Science, Universiti Putra Malaysia, Serdang 43400, Selangor Darul Ehsan, Malaysia; noorjahan@upm.edu.my
[6] Dr. Panjwani Center for Molecular Medicine and Drug Research, International Center for Chemical and Biological Sciences, University of Karachi, Karachi 75270, Pakistan; zaheer.qasmi@iccs.edu
[7] Research Institute of Natural Products for Drug Discovery (RiND), Faculty of Pharmacy, Universiti Teknologi MARA (UiTM), Puncak Alam Campus 42300 Bandar Puncak Alam, Selangor Darul Ehsan, Malaysia; benzene301@yahoo.com
* Correspondence: nadeemupm@gmail.com (M.N.A.); noorjahan@upm.edu.my (N.B.A.); Tel.: +60-9-549-2393 (M.N.A.); Fax: +60-9-549-2766 (M.N.A)

Received: 26 January 2018; Accepted: 21 February 2018; Published: 8 March 2018

Abstract: The biological activity of chalcone derivatives was predicted using PASS online server, which reveals the potential of flavokawain B type chalcones as caspase 3 stimulant, JAK2 expression inhibitor, and apoptosis agonist. Further molecular dynamics studies highlight the formation of stable contacts of these compounds within the active site of Janus Kinase.

1. Methods

To rationalize the anti-cancer potential of FKB derivatives, we have used an online web server PASS [1] to predict different biological activities. PASS server has been used by the researchers to predict biological activities of newly synthesized compounds [2] to rationalize the molecular basis of traditional practices of constituents of different plants [3] and to characterize bioactive natural products [4]. The experimental detailed of docking studies has been discussion in manuscript.

2. Computational Studies

To rationalize the observed anticancer activity of the newly synthesized chalcone derivatives, we have taken advantage of the Prediction of Activity Spectra for Substances (PASS), which is a free online server to predict the biological activity of the query compounds [3]. The server presents the results regarding the probability of activity (Pa) and inactivity (Pi). These predictions are based on the SAR analysis of more than 0.3 million compounds presenting more than 3750 different biological responses. Caspase 3 is one of chief effector caspases playing an indispensable role in the development biochemical and morphological hallmarks of apoptosis [4]. The activators of caspase-3 such as MX-2060 [4], Smac [5] and Leachianone A [6], induce apoptosis in various types of cancers. The high values of Pa of the compounds (0.892-0.924) allowed us to conclude that our newly

synthesized chalcone derivatives may exhibit anticancer effects by activating the caspase-3 dependent apoptotic pathway. Moreover, the cell specific response of the chalcone derivatives can be explained partly by the fact that the human MCF-7 breast carcinoma cells completely lack caspase-3 expression as a result of a frameshift mutation [7, 8]. This loss of function mutation has contributed to the chemotherapeutic resistance in breast cancer [4].

The accurate characterization of protein-ligand interactions is indispensable for the drug design. Molecular Dynamic Simulation is a state-of-art technique which is used to characterize the time-dependent behavior or a protein-ligand complex. The accurate characterization of protein-ligand interactions is indispensable for the drug design. Molecular Dynamic Simulation is a state-of-art technique which is used to characterize the time-dependent behavior or a protein-ligand complex. Herein, we have carried molecular dynamic simulation of the FKB derivatives complexed with the JAK2. The analysis of MD simulation allowed us to comprehend the time-dependent behavior of hydrogen which added potential insight into the dynamic behavior. In the case of FKB-1 complex, the observed hydrogen bonds between the ligand and the active site residues Lue855 and Gly856 were very unstable and were abrupt shortly.

Herein, we have carried molecular dynamic simulation of the FKB derivatives complexed with the JAK2. The average RMSD of all the four systems were recorded around 2 Armstrong (Figure S1, Table S1). Moreover, we did not notice any significant fluctuation in the atomic coordinates. These observations, taken together, pronounce the stability of our systems. The analysis of MD simulation allowed us to comprehend the time-dependent behavior of hydrogen which added potential insight into the dynamic behavior. In the case of FKB-1 complex, the observed hydrogen bonds between the ligand and the active site residues Lue855 and Gly856 were very unstable and were abrupt shortly. The initial and final binding mode of FKB1 complex presents significant deviation, which explains the observed volatile hydrogen bonding profile (Figure S2).

Table S1: The selected biological activities and their probabilities of activity (Pa) and (Pi) as obtained from PASS online server.

Name	Activity	Pa	Pi
	Caspase 3 stimulant	0.892	0.004
**1**	JAK2 inhibitor	0.850	0.005
	Apoptosis agonist	0.813	0.007
	Caspase 3 stimulant	0.924	0.003
**13**	JAK2 inhibitor	0.878	0.004
	Apoptosis agonist	0.821	0.007
	Caspase 3 stimulant	0.899	0.003
**15**	JAK2 inhibitor	0.859	0.004
	Apoptosis agonist	0.837	0.005
	Caspase 3 stimulant	0.906	0.003
**16**	JAK2 inhibitor	0.821	0.007
	Apoptosis agonist	0.827	0.006

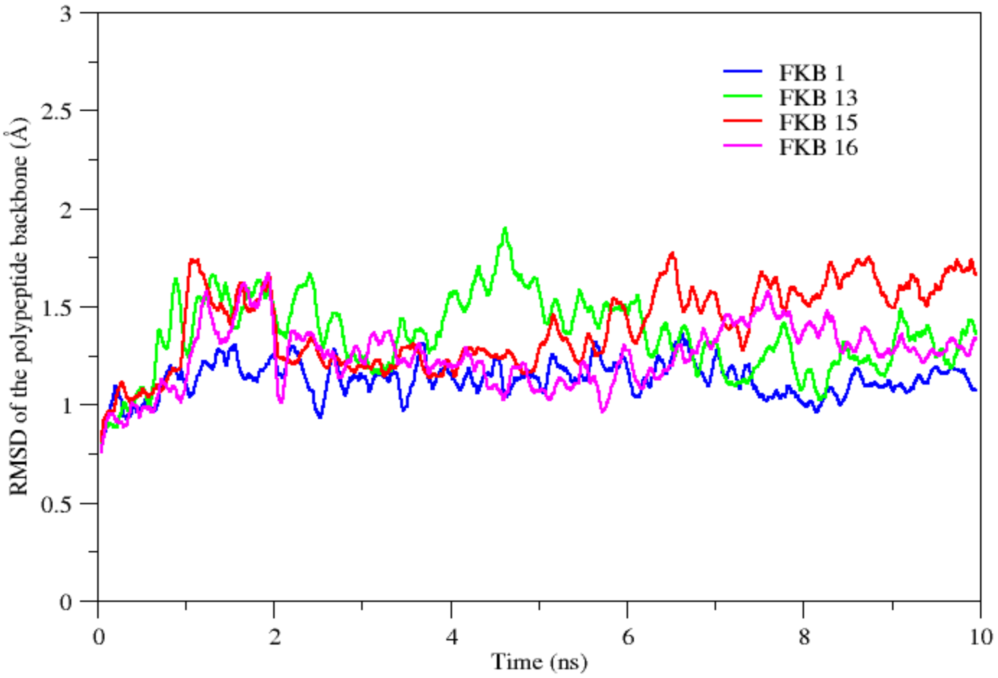

Figure S1: The graph presenting the trend of Root Mean Square Deviation (Ǻ) of all the four systems in the present study.

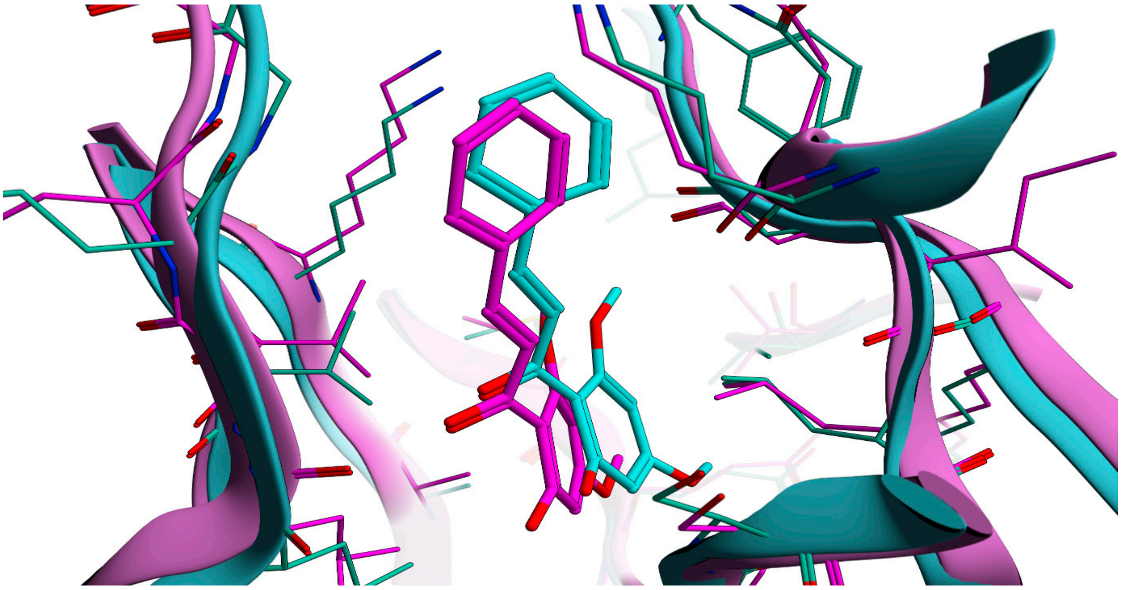

Figure S2: The difference in the initial and final coordinates of FKB1-JAK2 complex. The cyan color illustrates the initial pose (1ns) while the pose final (10ns) is presented in magenta. The ligand has presented significant deviation from the initial poses which explains the abrupt hydrogen bonding profile of the system.

References

1. Lagunin, A.; Stepanchikova, A.; Filimonov, D.; Poroikov, V. PASS: prediction of activity spectra for biologically active substances. *Bioinformatics*, **2000**, *16*, 747-748, doi:10.1093/bioinformatics/16.8.747.

2. Kadir, F.A.; Kassim, N.M.; Abdulla, M.A.; Yehye, W.A. Hepatoprotective Role of Ethanolic Extract of Vitex negundo in Thioacetamide-Induced Liver Fibrosis in Male Rats. *Evid. Based Complement. Alternat. Med.* **2013**, *2013*, 1-9, doi:10.1155/2013/739850.
3. Kadir, F.A.; Kassim, N.M.; Abdulla, M.A.; Yehye, W.A. PASS-predicted Vitex negundo activity: antioxidant and antiproliferative properties on human hepatoma cells-an in vitro study. *BMC Complement. Alternat. Med.* **2013**, *13*, 1-13.
4. Yang, X.H.; Sladek, T.L.; Liu, X.; Butler, B.R.; Froelich, C.J.; Thor, A.D. Reconstitution of Caspase 3 Sensitizes MCF-7 Breast Cancer Cells to Doxorubicin- and Etoposide-induced Apoptosis. *Cancer Research,* **2001**, *61*, 348–354.
5. Sun, H.; Nikolovska-Coleska, Z.; Lu, J.; Qiu, S.; Yang, C.Y.; Gao, W.; Meagher, J.; Stuckey, J.; Wang, S. Design, synthesis, and evaluation of a potent, cell-permeable, conformationally constrained second mitochondria derived activator of caspase (Smac) mimetic. *J. Med. Chem.* **2006**, *49*, 7916-7920.
6. Cheung, C.S.F.; Chung, K.K.W.; Lui, J.C.K.; Lau, C.P.; Hon, P.M.; Chan, J.Y.W.; Fung, K.P.; Au, S.W.N. Leachianone A as a potential anti-cancer drug by induction of apoptosis in human hepatoma HepG2 cells. *Cancer Letters,* **2007**, *253*, 224-235, doi:10.1016/j.canlet.2007.01.025.
7. Jänicke, R.U.; Sprengart, M.L.; Wati, M.R.; Porter, A.G. Caspase-3 Is Required for DNA Fragmentation and Morphological Changes Associated with Apoptosis. *J. Biol. Chem.* **1998**, *273*, 9357–9360.
8. Fulda, S.; Debatin, K.M. Caspase activation in cancer therapy. *Madame Curie Bioscience Database [Internet]*, **2013**, 1-29.

© 2018 by the authors. Licensee MDPI, Basel, Switzerland. This article is an open access article distributed under the terms and conditions of the Creative Commons Attribution (CC BY) license (http://creativecommons.org/licenses/by/4.0/).
